# The economic impact of the first wave of the pandemic on 50+ Europeans

**DOI:** 10.1007/s00181-022-02349-8

**Published:** 2023-01-18

**Authors:** Andrea Bonfatti, Greta Pesaresi, Guglielmo Weber, Nancy Zambon

**Affiliations:** 1 Padua University and Prometeia, Padua, Italy; 2 Padua University, Padua, Italy; 3 Padua University and IFS, Padua, Italy

**Keywords:** COVID-19, SHARE, Financial distress, Household economic conditions, D31, I32

## Abstract

We analyse the effects of the first wave of the COVID-19 crisis on the economic situation of 50+ Europeans. We construct a financial distress indicator that captures experiencing an income loss, difficulties to make ends meet and the need to postpone payments. We find that education and income before the pandemic has a protective role, and so does being past retirement age. For households under retirement age, instead, the pandemic has exacerbated inequalities. We also investigate whether households report worse difficulties in making ends meet compared to the pre-COVID-19 period. We show that their ability to make ends meet worsens more with income losses during the pandemic compared to losses experienced in the two-year period before the pandemic.

## Introduction

In this paper, we investigate the impact of the (first wave of the) COVID-19 pandemic on individuals aged 50 or more who live in 27 European countries or Israel by analysing changes in household income and various indicators of financial distress. We use recent SHARE (Survey of Health, Ageing and Retirement in Europe) data to identify the groups that have faced the most severe economic consequences, with potentially long-term implications, including the increased risk of poverty and social exclusion.

The COVID-19 pandemic has had a major impact on the lives and health of most individuals, on the economy in general, and the labour market in particular. One of the first policy reactions in many countries facing the outbreak of COVID-19 was to impose lockdown restrictions. The aim of those interventions was to reverse epidemic growth, reduce significantly severe case numbers, and stop the epidemic spread. Lockdown policies were successful in controlling the spread of the virus during the first wave of the pandemic, but also generated important economic consequences, unevenly distributed among different individuals.

Negative economic consequences are typical traits of recessions. However, recessions due to financial crises, such as the Great Recession, have different effects compared to pandemic recessions. Firstly, in the Great Recession, all age groups, education levels, and income quintiles experienced income declines (De Nardi et al. 2012). Secondly, many households were adversely affected by the Great Recession even if their income did not change, as the value of their homes or retirement savings plummeted (Meyer and Sullivan 2013). On the other hand, lessons from the Great Recession that are relevant to the pandemic are that job losses have persistent effects on employment and income for older workers who are less likely to find a job similar to their previous one and may be forced to opt for early retirement (Bui et al. 2020; Li and Mutchler 2020).

We show that the pandemic is leading to increased economic inequality in the 28 countries we consider. This is not surprising, because less educated and less well-paid workers are more vulnerable to income losses and lay-offs (ILO 2020; Stiglitz 2020), while working from home is more easily available to the better paid, better educated workers (Deaton 2021). The impact of the COVID-19 pandemic also depends on country characteristics (Fana et al. 2020): countries that rely on service activities, such as Mediterranean countries, are more likely to suffer.

Understanding the economic and social costs of the pandemic, ranging from job losses to shuttered businesses, is of critical importance to develop effective and sustainable policies. Our focus on 50+ Europeans allows us to draw the important distinction between older workers and retirees—where the former are directly exposed to labour market risks and the latter should in principle be insured by the pension system.

We investigate the relation between economic effects of the COVID-19 crisis and various socio-demographic, economic, and employment indicators. We contribute to the literature in several dimensions. First, we investigate and document the effects of household type (singles versus couples), age, education, income, employment, and policy interventions on financial distress. Second, we propose a new comprehensive measure of household financial distress.

The econometric analysis of our financial distress indicator highlights the protective role of education and income before the onset of the pandemic. We also find that those who did not report difficulties in making ends meet in the past were less likely to be in financial distress during the pandemic. We find that employment-related events (such as job loss or reduced working hours) are an important channel through which the pandemic negatively affected household economic conditions. The possibility to work from home instead reduced financial distress. Taken together, these results confirm that the pandemic had a milder effect on the better off, thus exacerbating economic inequality (at least among working-age individuals, aged 50 or more).

We also investigate variations in the ability to make ends meet between the wave 7 of SHARE (run in 2017–2018) and the first SHARE Corona Survey (run in June–September 2020). We show that while age has again a protective role, the ability to make ends meet worsens with either an income loss due to the COVID-19 crisis or more generally an income loss across waves. The increase in the probability of a worsening due to losses during the pandemic is five times larger than the increase induced by losses across waves. The level of income before the outbreak of COVID-19, instead, retains its protective role. We find that employment conditions and their variations (before and during the pandemic) have little or no effect on the probability of a deterioration in the ability to make ends meet. Among other sources of income, real and financial investments reduce the probability of a worsening, while owning a business increases it. We find that being a tenant and the length of governmental restrictions increase the probability of financial distress. The same variables affect improvements in ability to make ends meet but in the opposite direction. We observe that, in the latter case, having a partner has a no effect or negative effect on the probability of improvement.

An important finding of our analysis is that individuals past retirement age are less likely to be in financial distress or to face increased difficulties in making ends meet with respect to 50+ individuals below retirement age, and this confirms that the European public pension (social security) systems have been successful in protecting older individuals. Income support measures for younger individuals (aged 50 or more but below retirement age) do not seem to have worked as well, instead.

The paper is organized as follows. Section 2 presents the data; Sect. 3 shows descriptive statistics while Sect. 4 reports regression results. Section 5 concludes.

## The data

We use data drawn from the Survey of Health, Ageing and Retirement in Europe (SHARE), a longitudinal, multidisciplinary, and cross-national European dataset. The dataset includes current and retrospective information on health, socio-economic status and social and family networks of individuals aged fifty or older in (currently) twenty-seven European countries (plus Israel). We use data from the first SHARE Corona Survey, or SCS, (Börsch-Supan 2022) that complements the regular wave 8 (Scherpenzeel et al. 2020), plus information from older waves when necessary. This allows us to account for different detailed characteristics, at the individual or household level, and highlights heterogeneous economic consequences of the epidemic related to prior conditions.

We only partially use data from the regular wave 8 as face-to-face data collection was suspended in March 2020 due to the COVID-19 outbreak. Shortly after the COVID-19 outbreak, a new telephone administered survey, the SCS, was introduced with the aim to collect data on health and socio-economic impacts of COVID-19 among SHARE respondents. The data collection started in June and ended for all countries but Austria in August 2020. Fieldwork in Austria, instead, ran from July till September 2020. We checked that the exclusion of Austria does not affect our results.

Our sample includes individuals aged 50 or more (and their spouses or partners) living in twenty-seven European countries, namely Austria, Belgium, France, Germany, Luxembourg, Switzerland, Sweden, Denmark, Finland, Spain, Italy, Greece, Portugal, Cyprus, Malta, Netherlands, Estonia, Latvia, Lithuania, Poland, Czech Republic, Slovakia, Hungary, Bulgaria, Romania, Slovenia, and Croatia, plus Israel.

In the SCS participants were asked to report, among other things, the economic and working conditions before and during the pandemic. Since we are interested in investigating the impact of the first wave of the pandemic on household economic inequality, we focus on a subset of this information.

For the economic aspects we use information about the ability to make ends meet (both in wave 7 and in SCS), the lowest household income, the need to postpone payments or dip into savings during the pandemic, but also on the household typical monthly income before the pandemic.^1^ The ability to make ends meet is a widely used indicator of the general financial conditions (e.g. Saunders et al. 1994), in which individuals evaluate their conditions with respect to their household needs. In SHARE, this subjective measure is evaluated on an ordered scale with four response options: with great difficulty, with some difficulty, fairly easily, and easily. We define a binary indicator for households with some or great difficulties. For households reporting difficulties, there are two follow-up questions regarding (1) the need to postpone regular payments such as rent, mortgage and loan payments, and/or utility bills and (2) the need to dip into savings to cover necessary day-to-day expenses.

As regards the working conditions we use information on the employment situation before the pandemic (reported both in wave 7 and in SCS), and employment conditions since its outbreak: place of work (home and/or usual workplace); potential job interruptions due to unemployment, lay-off or business closure; and reduction of working hours.^2^

Questions on household income and the ability to make ends meet were asked also in previous regular waves. This allows us to take a longitudinal perspective, which is a peculiar characteristic of SHARE.

The data allow us not only to have a broad perspective on the economic impact of the first wave of the pandemic on European working-age (between 50 and 64) and retirement-age (over 64) households, but also to investigate and highlight differential effects among countries.

We restrict our sample to respondents answering the SCS and taking part to the last publicly available wave (wave 7). In our final sample, there are 50,437 individuals participating to the SCS and observed in wave 7 (for whom we have a longitudinal perspective), plus 7122 individuals participating only in the COVID-19 survey. Our economic outcomes of interest are defined at the household level: in our sample, we observe 39,104 households.^3^ However, we restrict our sample to the 31,227 households (45,479 respondents) for which we have all information needed for our analyses, either reported by the respondents or imputed.^4^ As of July 2022, the available SHARE imputations still suffer from noticeable outliers that may have high leverage on the analysis of the complete data. To overcome this issue, we produced our own imputations for the set of variables that are relevant in our analyses. We refer the reader to Appendix 6.1 for more details on the imputation process.^5^ All results, both weighted descriptive statistics and estimation results, are adjusted to account for the variability between imputations using Rubin’s combination rule (Rubin 1987). Thus, standard errors of the mean are computed correcting for the multiple imputations’ component.

Table 1 reports the weighted summary statistics for the outcomes of interest in our sample of 50+ respondents and their spouses/partners. Household income is in Euros per month. The average value of the household typical monthly income before the pandemic is €2158 (standard error 13.17). The mean value of the lowest overall monthly income, after taxes and contributions, that households report during the pandemic is, instead, €2050 (standard error 11.67). This suggests that, on average, the drop in income has been moderate (5%), but this is in-line with the fact that a large fraction of sample respondents are pensioners who rely on pensions or other social protection benefits for older persons. If we split the sample between ‘working-age’ and ‘retirement-age’ households, where the former households have at least one member under 65 in the couple, we can see that the average income drop among the former is 6.88% compared to 2.61% among the latter. This is prima facie evidence that social security systems have effectively protected older individuals. Moreover, Table 1 reports the percentage of households that experienced an income loss of at least 5% during the pandemic (i.e. the lowest household income during the pandemic is at least 5% lower than the typical household income before the outbreak of the pandemic.). In-line with the similar amounts of typical and lowest overall monthly incomes in Table 1, only 17.52% of households experienced an income loss. It is worth noting that the lowest monthly income includes financial support households may have received (from government, employer, relatives, friends, or others) and, thus, the limited income loss may reflect the efficacy of government policies in contrasting the negative economic consequences of the pandemic on household incomes.

**Table 1:** Summary statistics (SCS)—household economic and employment outcomes

Outcomes	Mean	SE	Obs
Typical overall monthly income SCS	2158.18	13.17	31,227
At least one HH member under 65	2508.63	21.05	9210
All HH members 65+	1833.41	14.55	22,017
Lowest overall monthly income	2050.32	11.67	31,227
At least one HH member under 65	2335.98	20.00	9210
All HH members 65+	1785.59	13.64	22,017
Typical overall monthly income Wave 7	2294.47	11.46	31,227
At least one HH member under 65	2617.36	21.18	9210
All HH members 65+	1995.24	13.29	22,017
Income loss SCS	17.52%	0.38%	31,227
At least one HH member under 65	25.83%	0.58%	9210
All HH members 65+	9.82%	0.42%	22,017
Difficulties in Making ends meet	30.87%	0.30%	31,227
Postpone payments	11.91%	0.33%	10,555.6
Dip into savings	26.60%	0.48%	10,555.6
Financial Distress Indicator (FDI)	0.50	0.004	31,227
High Financial Distress (FDI = 3)	1.92%		
Mild Financial Distress (FDI = 2)	7.83%		
Low Financial Distress (FDI = 1)	30.65%		
No Financial Distress (FDI = 0)	59.60%		
Employment	36.90%	0.27%	31,227
Job interruption	21.81%	0.48%	7357
Number of weeks of job interruption	8.82	0.12	1604.8
Work from home	35.19%	0.56%	7357
Reduction in working hours	20.79%	0.48%	7357

The table shows household descriptive statistics (mean, standard error of the mean, and number of observations), using calibrated cross-sectional household weights and multiple imputations, of economic and employment outcomes in the SCS and in wave 7 (only typical income). Incomes, typical and lowest, are in Euros and represent, respectively, the typical household income in 2017 (wave 7) and before COVID-19 broke out (SCS), and the lowest household income during the first wave of the pandemic. For each income variable, the table also shows summary statistics for households with at least one member younger than 65, and for households with all members 65 or more. Income loss SCS is a binary variable that takes value one if the household reports an income loss during the first wave of the pandemic. We consider an income loss when the lowest household income during the pandemic is at least 5% lower than the typical household income before the outbreak of the pandemic. Difficulties in making ends meet is a binary indicator, taking value one if the household reports having had some or great difficulties in making ends meet since the outbreak of COVID-19. Postpone payments and dip into savings variables record whether households with difficulties in making ends meet postponed payments (such as rent, mortgage and loan payments, and/or utility bills) and/or dipped into savings to cover day-to-day expenses. FDI is the Financial Distress Indicator. It reflects, using a score between 0 (no distress) and 3 (high distress), the negative financial effect of the pandemic on households. Employment is a binary variable that equals one if there was at least one employed household respondent at the time when COVID-19 broke out. Job interruption and work from home are employed households with at least one respondent who, respectively, experienced job interruption and worked from home (both work from home only, as well as work from home and at the usual workplace) during the pandemic. Number of weeks of job interruption is a follow-up question in case someone in the household reports a job interruption and equals the maximum among household respondents. Reduction in working hours is a variable reflecting reduced working hours for households with at least one employed respondent who did not experience job interruptions during the pandemic. For variables that are collected conditional on difficulties making ends meet (2 variables) and being in employment (4 variables), imputations are computed only when the condition is met, and the average number of observations can be not an integer

*Source* Authors’ elaboration using SCS and SHARE waves 1, 2, 4, 5, 6, 7, and 8 data

Households experienced an average income loss also between wave 7 and the SCS. Typical income decreased by 5.77%, but it was much larger for ‘retirement-age’ households, 8.11%, than for ‘working-age’ households, 4.15%.

Table 1 highlights that in our sample the fraction of households reporting (some or great) difficulties in making ends meet is about 30%. Among those, 11.91% had to postpone payments and 26.60% used their savings to cover necessary day-to-day expenses. It is worth noting that among the possible answers to the dip-into-savings question, there is not the option “had no savings”. Therefore, among households who report failing to dip into savings there will be some who chose not to use them and others who could not use them because they did not have any.

We define a Financial Distress Indicator (FDI) that reflects the negative financial effect of the pandemic on households. The indicator is the sum of three dummy variables: income loss (during the pandemic), difficulties in making ends meet (during the pandemic), and (conditional on experiencing difficulties) postponed payments. The Financial Distress Indicator measures, using a score between 0 and 3, the severity of the economic difficulties suffered by households during the pandemic. In Table 1 we report the average value of the FDI (0.50, a relatively low number) and the proportion of households characterised by High Financial Distress (FDI = 3, less than 2%), Mild Financial Distress (FDI = 2, around 8%), Low Financial Distress (FDI = 1, 31%) and No Financial Distress (FDI = 0, almost 60%).

Policy interventions introduced in many countries aimed at containing the spread of the virus affected asymmetrically individuals and households depending on their predetermined characteristics. A key role was played by the employment status. Incomes from pensions were generally unaffected; labour income and incomes from other sources could experience a sharp drop since the outbreak of COVID-19, depending, among other factors, on the occurrence of job interruption, the possibility to work remotely and/or on the reduction of the working hours. We can see from Table 1 that 36.90% of the households were employed at the time COVID-19 broke out. Among them, 21.81% experienced at least one job interruption due to unemployment, lay-off or business closure, with an average number of interruption weeks of 8.82, and 35.19% worked, at least partly, from home. In the subsample of households with at least one employed partner who did not experienced job interruptions, 20.79% reduced their working hours during the pandemic.

Table 2 describes our sample of 50+ Europeans in terms of socio-demographic characteristics: age, gender, marital status, household size and education (expressed according to the International Standard Classification of Education—ISCED).

**Table 2:** Summary statistics (SCS)—socio-demographic characteristics

Characteristics	Mean	SE	Obs
Maximum age	69.42	0.056	31,227
Minimum age	67.34	0.060	31,227
Gender—female	85.92%		31,227
Marital status—couple	59.66%		31,227
Household composition			31,227
Single younger than 65	12.57%		
Single 65 or older	27.77%		
Couple with at least one member under 65	35.53%		
Couple with all members 65+	24.13%		
Household size	2.13	0.006	31,227
Education			31,227
Primary	9.43%		
Lower secondary	14.15%		
Upper secondary	42.95%		
Post-secondary non-tertiary	12.45%		
Tertiary	21.02%		

The table shows descriptive statistics (mean, standard error of the mean, and number of observations), using calibrated cross-sectional household weights and multiple imputations, of SCS respondents’ socio-demographic characteristics. Age, and education refer to, respectively, maximum/minimum age and maximum educational attainment between household respondents. Gender is a binary indicator, taking value one if there is at least one female between the respondents. Marital status is a binary indicator that equals one if household respondents are in a couple, and zero in case of a single household respondent. Household composition classifies households in singles or couples and, among them, according to age. Household size takes values between 1 and 11

*Source* Authors’ elaboration using SCS and SHARE waves 1, 2, 4, 5, 6, 7, and 8 data

We can see from Table 2 that the average maximum and minimum age (within household respondents) are, respectively, 69.42 (standard error 0.056) and 67.34 (standard error 0.060). The sample is composed by 85.92% households with at least one female between the respondents; 12.57% are single under-65 respondents, 27.77% are single over-64 respondents, 35.53% are couples with at least one member younger than 65, and 24.13% are couples with all members older than 64. The average household size is 2.13 with a standard error of 0.006. The majority (78.98%) of households have a medium–low level of education (primary to post-secondary).

Finally, we complement the analysis exploiting data from the Oxford COVID-19 Government Response Tracker (OxCGRT). The OxCGRT collects information on several common policy interventions that governments implemented to respond to the pandemic. We use information about the strictness and length of ‘lockdown style’ closures and containment policies (the so-called “stringency index”).^6^ We use weekly means of daily values of the stringency index from January 2020 until September 2020. Our aim is to capture the intensity of, and the period covered by policy interventions and their potential economic consequences for different individuals.

## Variables of interest: descriptive statistics

This section presents a descriptive analysis of economic outcomes during the first wave of the pandemic for different subsamples of the population of interest. The aim is to highlight, in our sample of individuals aged 50 or more, the most relevant household characteristics associated to severe economic consequences due to the pandemic.

### Employment and economic outcomes

Participation in the labour market by working-age individuals is obviously important in many respects, not least for their contribution to the overall household income. In this section, we assess the role of COVID-19-related job interruptions (due to unemployment, lay-off or business closure) and reduction of working hours on household economic conditions.

Focusing on employed households, we define households with job interruption as households in which at least one respondent, who was employed before the pandemic, experienced one or more job interruptions during the pandemic.^7^

Table 3 describes our subsample (7357 employed households) according to occurrence of job interruption. Table 3 shows that 21.81% of households experienced a job interruption. However, job interruption shows great variability among countries.

**Table 3:** Summary statistics (SCS)—job interruptions

Country	Mean (%)	SE (%)	Obs	Country	Mean (%)	SE (%)	Obs	Country	Mean (%)	SE (%)	Obs
Austria	37.70	3.41	203	Belgium	29.39	1.83	620	Lithuania	22.28	2.61	255.2
Germany	19.87	1.70	554	Israel	24.82	2.92	221.8	Bulgaria	24.14	3.67	137
Sweden	12.33	2.27	210	Czech Rep	12.89	1.71	383.4	Cyprus	46.27	9.42	29
Netherlands	2.84	2.11	63	Poland	5.45	1.22	347	Finland	21.02	2.69	230
Spain	29.67	4.15	122	Luxembourg	42.12	5.77	74.2	Latvia	6.68	2.16	134.2
Italy	33.66	2.32	414	Hungary	10.18	3.51	75	Malta	14.82	4.07	77.4
France	44.63	3.26	233.8	Portugal	34.39	4.23	127	Romania	17.47	3.44	123
Denmark	8.47	1.20	536.8	Slovenia	43.48	3.34	220	Slovakia	19.22	2.72	211
Greece	37.15	3.00	261	Estonia	13.60	1.12	934.8				
Switzerland	30.39	2.41	366.4	Croatia	10.66	2.27	193	Total	21.81	0.48	7357

The table shows descriptive statistics, using calibrated cross-sectional household weights and multiple imputations, of job interruptions during the first wave of the pandemic due to unemployment, lay-off or business closure. For each country, the table shows the number of employed households at the time pandemic broke out (Obs.), which are households with at least one employed respondent, the fraction of them that experienced at least one job interruption during the pandemic (Mean), that is households with at least one employed respondent that experienced one or more job interruptions, and the corresponding standard errors of the mean (SE). Since the variable job interruptions is collected conditional on being in employment, imputations are computed only when the condition is met, and the average number of observations can be not an integer

*Source* Authors’ elaboration using SCS and SHARE waves 1, 2, 4, 5, 6, 7, and 8 data

Job interruptions may be a channel through which the pandemic negatively affected household economic conditions. Indeed, job interruptions may cause an income loss and, consequently, may lead to financial distress. In the whole subsample of employed households with an income loss, 48.18% also experienced at least one job interruption. However, income loss cannot be explained by the occurrence of job interruptions in the remaining 51.82% of the subsample. Focusing on employed households without job interruptions but with income losses, 44.49% of them report a reduction of working hours. Alternative possible explanations for income losses lie in the reduction of other sources of income.^8^

### The role of education and age

The pandemic and the consequent government interventions had a heterogeneous economic impact on individuals and households depending on their predetermined characteristics, and, among them, a key role was played by socio-demographic types. The literature has widely investigated the role of education, age, and marital status on economic outcomes. In this section, we investigate the link between household education, age, and type (single vs. couple), and the economic impact of the pandemic, to shed light on their role in mitigating negative economic outcomes.

Table 4 shows, for each household educational level, the percentage of households reporting no, low, mild, and high financial distress during the first wave of the pandemic. We measure financial distress using a categorical Financial Distress Indicator (FDI).

**Table 4:** Household level of financial distress by education, %

	Primary (or none)	Low secondary	Upper secondary	Post-secondary	Tertiary
High financial distress	0.72	1.64	2.45	1.68	1.71
Mild financial distress	10.31	9.78	8.37	5.91	5.44
Low financial distress	43.57	44.05	30.53	24.45	19.73
No financial distress	45.40	44.53	58.64	67.96	73.12
Obs	2944	4420	13,411	3887	6565

The table shows the percentage of households that report no, low, mild, and high financial distress during the first wave of the pandemic (SCS) by household level of education (i.e. the level of education of either the respondent—single household—or the most educated couple respondent), using calibrated cross-sectional household weights and multiple imputations. Financial distress is measured using a score between 0 and 3. It reflects the severity of the economic difficulties suffered by households during the pandemic

*Source* Authors’ elaboration using SCS and SHARE waves 1, 2, 4, 5, 6, 7, and 8 data

From Table 4 we learn that households reporting distress are asymmetrically distributed among educational levels. Households with low education (primary and lower secondary) were more affected by distress compared to household characterized by medium–high education (secondary and, especially, tertiary). These results suggest a protective role of education on financial distress (and on the worsening of the ability to make ends meet, see Appendix 6.2) during the first wave of the pandemic.

We now investigate the role of age and household type on economic outcomes. Table 5 shows a protective role of both age and having a partner on financial distress during the pandemic. Households who are 65 older report less distress compared to their younger counterparts (single and couples under 65). We can draw similar conclusions for having a partner. Single households report more financial distress than couples.

**Table 5:** Household financial distress and age, %

	Single < 65	Single 65+	Couple < 65	Couple 65+
High Financial Distress	3.66	0.49	3.80	0.47
Mild Financial Distress	13.48	6.44	11.30	3.96
Low Financial Distress	39.05	36.74	31.60	25.55
No Financial Distress	43.81	56.33	53.30	70.01
Obs	3924	8672	11,096	7535

The table shows the percentage of households that report no, low, mild, and high financial distress during the first wave of the pandemic (SCS) by household composition defined according to type and age (i.e. single vs. couple, under 65 vs. 65 or over), using calibrated cross-sectional household weights and multiple imputations. Couple < 65 consists of couples in which at least one member is younger than 65, while couple 65+ comprises couples with both members older than 64. Financial distress is measured using a score between 0 and 3. It reflects the severity of the economic difficulties suffered by households during the pandemic

*Source* Authors’ elaboration using SCS and SHARE waves 1, 2, 4, 5, 6, 7, and 8 data

Table 6, instead, shows the role of real estate, main residence and “second homes”, in reporting a worsening in making ends meet between wave 7 and the first wave of the pandemic (SCS). Here we consider a subsample of households who can report a worsening in making ends meet, thus we restrict our attention on households with no difficulties in wave 7. We can see from Table 6 that owners of main residence (only for under 65 households) and second homes report less often a worsening in ability to make ends meet. Possible drivers of this result could be a general higher overall wealth of homeowners, and the additional income flow from second homes. Moreover, results in Table 6 confirm those of Table 5, as the percentage of households who report increased difficulties is lower among those who are 65+. Thus, in this respect too, age plays a protective role.

**Table 6:** Percentage of households with a worse economic situation by owning real estate (main residence or second homes) and age

	Own main residence		Tenant	
	At least one HH member under 65	All HH members 65+	At least one HH member under 65	All HH members 65+
*Main residence* (*a*)				
Worse MeM	14.78	11.88	18.15	11.72
Not Worse MeM	85.22	88.12	81.85	88.28
Obs	4650	11,265	620	1802

	Has Second Home		No Second Home	
	At least one HH member under 65	All HH members 65+	At least one HH member under 65	All HH members 65+
*Second home* (*b*)				
Worse MeM	13.83	9.30	15.90	12.69
Not Worse MeM	86.17	90.70	84.10	87.31
Obs	1753	3515	3517	9552

The table shows the percentage of households that worsened the economic condition, measured by a worsening in making ends meet ability between wave 7 (2017) and the first wave of the pandemic (SCS), by age and either by main residence ownership (part a) or second home ownership (part b), using calibrated cross-sectional household weights and multiple imputations. This table refers to a subsample of households that did not report some or great difficulties in making ends meet in 2017

*Source* Authors’ elaboration using SCS and SHARE waves 1, 2, 4, 5, 6, 7, and 8 data

## Estimation results

In this section, we present our estimation results, for our sample of individuals aged 50 or more, when the dependent variable is a measure of household economic conditions during the first wave of the pandemic. Our dependent variables are a binary indicator for self-reported difficulties to make ends meet and a categorical Financial Distress Indicator (FDI).^9^ We stress that we do not identify causal effects, rather partial correlations.

In Table 7 we report the ordinary least squares (OLS) estimates of key parameters of the models that explain the ability to make ends meet during the pandemic (“MeM_SCS_”)—in this case we are estimating a linear probability model—and the “FDI” as a function of socio-demographic, economic and employment household characteristics, plus contextual information on COVID-19-related policy interventions introduced by governments.^10^

**Table 7:** OLS regressions—dependent variables: difficulties in making ends meet (SCS) and FDI

	MeM_SCS_	FDI
Female	− 0.003	− 0.019*
	(0.006)	(0.010)
Household size	0.021***	0.042***
	(0.003)	(0.005)
*HH composition and age*		
Single ≥ 65	− 0.082***	− 0.151***
	(0.009)	(0.015)
Couple < 65	0.020*	− 0.033*
	(0.010)	(0.018)
Couple ≥ 65	− 0.035***	− 0.131***
	(0.010)	(0.016)
Years of education	− 0.004***	− 0.003**
	(0.001)	(0.001)
Employed	− 0.006	0.071***
	(0.008)	(0.014)
Job interruption	− 0.016	0.232***
	(0.021)	(0.044)
Home working	− 0.016*	− 0.067***
	(0.009)	(0.016)
Reduced working hours	0.024**	0.232***
	(0.011)	(0.022)
Weeks of job interruption	0.013***	0.033***
	(0.002)	(0.004)
Income from others	0.009*	0.017*
	(0.006)	(0.009)
Second homes	− 0.011**	− 0.013
	(0.005)	(0.008)
Investments	− 0.033***	− 0.043***
	(0.005)	(0.009)
Own business	0.039***	0.116***
	(0.009)	(0.015)
Tenant	0.049***	0.072***
	(0.007)	(0.011)
log(Income before COVID-19)	− 0.225***	− 0.237***
	(0.006)	(0.010)
Length of restrictions	0.002	− 0.001
	(0.001)	(0.002)
Avg. restriction intensity	− 0.001	− 0.001
	(0.004)	(0.006)
MeM wave7	0.246***	0.283***
	(0.006)	(0.009)
Observations	31,227	31,227
*R*^2^	0.397	0.337

Dependent variable in columns 1 is a binary indicator for (some or great) difficulties in making ends meet during the pandemic, while dependent variable in column 2 is the Financial Distress Indicator. The latter variable reflects, using a score between 0 and 3, the negative financial effect of the pandemic on households. The results obtained using multiple imputation technique for missing values. Robust standard errors in parentheses. ***p  < 0.01; **p < 0.05; *p < 0.1

*Source* Authors’ elaboration using SCS and SHARE waves 1, 2, 4, 5, 6, 7, and 8 data

We use the following household-level controls: country dummies, age, gender, household size and type (i.e. single vs. couple), education, employment-related variables (e.g. occurrence of job interruptions and reduction in working hours), other sources of income (e.g. income from other household members, and businesses), being tenant/subtenant, income before COVID-19 crisis, length and intensity of restrictions.^11^

Estimation results with different dependent variables (income loss—“IncLoss_scs_”, postponed payments—“Postpay”, and dipped into savings—“DipSav”) are presented in Appendix 6.3. Note that results when the dependent variable is the “FDI” are, in general, in-line with the results we obtain using, in turn, difficulties in making ends meet, income loss, and postponed payments as dependent variables.

The results in Table 7 column 1 show that older age plays a protective role on ability to make ends meet (MeM_SCS_), as both coefficients on “single ≥ 65” and “couple ≥ 65” are negative and significant. “Years of education” and the income level before the outbreak of the pandemic also play a protective role. Having a partner and being in a household with at least one couple respondent younger than 65, instead, increases the probability of reporting difficulties in making ends meet (positive and significant coefficient on “couple < 65”). As regard employment-related variables, while coefficients on “Reduced working hours” and “Weeks of job interruption” are positive and significant indicating that a reduction of work during the first wave of the pandemic increased the probability of reporting difficulties, “Home working” reduces the probability (negative and significant coefficient). Lastly, the coefficient on Make-ends-meet wave 7 (“MeM wave7”) is positive and significant, indicating that difficulties show persistency.

The results in Table 7 column 2 show results that are in-line with the results in column 1. Coefficients on “single ≥ 65”, “couple ≥ 65”, “Year of education”, and “log(Income before COVID-19)” are negative and significant, confirming the protective role of older age, education, and income. This suggests that the pension systems successfully insured the retired against the shock, while specific government policies for younger households did not fully offset the negative effects of the pandemic. Differently from results in column 1, here also having a partner decreases the probability of financial distress. Among working-age households, the shock hit harder already vulnerable households, with low economic resources. Estimated coefficients for employment-related variables (“Employed”, “Job interruption”, “Reduced working hours”, “Weeks of job interruption”, and “Home working”) show greater financial distress for households with employed individuals, and, in particular, for those who experienced job interruptions or reductions in working hours, and could not work from home. Thus, less educated and less well-paid workers were not only more exposed to income losses and lay-offs (ILO 2020; Stiglitz 2020), but are also more likely to experience financial distress.

It is also worth stressing that, also in this case, the coefficient on “MeM wave7” is positive and significant, indicating that difficulties show persistency. This confirms that the pandemic exacerbated economic inequalities, and ad hoc governmental measures were unable to protect the poorer.

Longitudinal data in our dataset allow us to study how household economic distress changed through time. Respondents provided information on their ability to make ends meet and on their income both in wave 7 and in the SCS. We can thus investigate which factors affect the probability to report a worsening (“MeM worsening”) or an improvement (“MeM improvement”) in make ends meet ability.

Table 8 shows the estimates of two OLS regressions when the dependent variable is either “MeM worsening” or “MeM improvement”. For the former, we consider the subsample of households who could report a worsening in the SCS (thus, households without difficulties in making ends meet in wave 7); for the latter, we focus on the subsample of households who could experience an improvement (households with difficulties in wave 7).

**Table 8:** OLS—dependent variables: worsening and improvement in making ends meet

	MeM_worsening	MeM_improvement
Female	− 0.009	− 0.017
	(0.007)	(0.012)
Household size	0.016***	− 0.022***
	(0.004)	(0.005)
plusDeltaSize	− 0.004	− 0.008
	(0.023)	(0.028)
minDeltaSize	0.021**	0.010
	(0.009)	(0.013)
*HH composition and age*		
single ≥ 65	− 0.074***	0.075***
	(0.012)	(0.014)
couple < 65	0.000	− 0.044***
	(0.013)	(0.017)
couple ≥ 65	− 0.038***	0.023
	(0.013)	(0.016)
Years of education	− 0.004***	0.003**
	(0.001)	(0.001)
*Employment variation:*		
Always employed	− 0.006	0.023
	(0.011)	(0.017)
Employed-not empl	0.013	− 0.012
	(0.010)	(0.017)
Not empl—employed	0.026*	0.022
	(0.015)	(0.025)
Job interruption	− 0.048*	− 0.035
	(0.026)	(0.040)
Home working	− 0.014	0.060***
	(0.009)	(0.023)
Reduced working hours	0.003	− 0.032
	(0.012)	(0.024)
Weeks of job interruption	0.011***	− 0.012***
	(0.003)	(0.004)
IncLoss_SCS	0.095***	
	(0.011)	
IncLoss_waves	0.019***	
	(0.006)	
IncGain_SCS		0.049**
		(0.019)
IncGain_waves		0.021**
		(0.009)
log(Income before COVID-19)	− 0.171***	0.274***
	(0.008)	(0.011)
Income from others	0.008	− 0.011
	(0.006)	(0.010)
Second homes	− 0.014**	0.012
	(0.006)	(0.010)
Investments	− 0.023***	0.093***
	(0.005)	(0.017)
Own business	0.036***	− 0.052***
	(0.009)	(0.020)
Tenant	0.032***	− 0.080***
	(0.008)	(0.012)
Length of restrictions	0.003*	− 0.001
	(0.002)	(0.003)
Avg. restriction intensity	0.001	0.005
	(0.004)	(0.008)
Observations	18,337	12,890
*R*^2^	0.199	0.237

The table-dependent variable in column 1 is a binary indicator for worsening in making ends meet from wave 7 to SCS, for the subsample of households that did not report some or great difficulties in making ends meet in wave7. Dependent variable in Column 2, instead, is a binary indicator for improvement in making ends meet from wave 7 to SCS, for the subsample of households who had some or great difficulties in wave7. The results obtained using multiple imputation technique for missing values. Robust standard errors in parentheses. ***p  < 0.01; **p < 0.05; *p < 0.1

*Source* Authors’ elaboration using SCS and SHARE waves 1, 2, 4, 5, 6, 7, and 8 data.

We control for country, age, gender, household size (level and changes) and type, employment-related variables (both in wave 7 and in the SCS), education, dummies for income loss/gain between waves *(typical income before pandemic outbreak—typical income in wave 7)* and during the pandemic *(lowest income during the pandemic—typical income before pandemic outbreak)*, income before COVID-19 crisis, other sources of income (e.g. income from other household members, and businesses), being tenant/subtenant, length and intensity of restrictions.^12^

The results in Table 8 column 1 confirm a protective role of older age, income prior to the pandemic, and education. The coefficients on income loss variables, both between surveys—“IncLoss_waves”—and during the pandemic—“IncLoss_SCS”—are positive and significant, implying a higher probability of experiencing worse difficulties in making ends meet. Income losses during the pandemic were much more important than losses between waves, with coefficients that equal, respectively, 0.095 and 0.019.

Given the prominent role of income variables, we investigate which other factors affect the variation in ability to make ends meet for households with and without income losses/gains (see Appendix 6.3). We find that, for households who experienced at least an income loss, “IncLoss_SCS” significantly increases the probability of a worsening, while the coefficient for “IncLoss_waves” is not significant. These results confirm that household worsening to make ends meet reacts more to income losses during the pandemic than to losses between waves. Employment and economic household variations between waves cover a long-time span but have little or no impact on the probability of a worsening. COVID-19-related variations, instead, affect the probability of a worsening despite referring to a more recent but shorter time window.

## Conclusions

In this paper, we have investigated the economic effects of the first wave of COVID-19 crisis on households, using several indicators of financial distress. Our rich dataset on 50+ Europeans, which includes longitudinal data and data from the first SHARE Corona Survey (run in June–September 2020), allows us to identify the groups that have faced the most severe economic consequences.

COVID-19 had heterogeneous effects on the population, not only in terms of health and mental health [see among others Angelucci et al. (2020), Adams-Prassl et al. (2022), Bertoni et al. (2021a, b)], but also in terms of financial distress. Indeed, we find heterogeneous economic consequences faced by households that depend on demographic characteristics, age, and household type, as well as on income and employment conditions before and during the pandemic. Using a new comprehensive financial distress indicator (FDI), we show that 65+ households were less affected by financial distress (with respect to individuals aged 50–64), indicating an efficient protection of individuals past retirement age by social security systems. For working-age individuals, instead, employment conditions changed because of governmental restrictive measures aimed at reducing the spread of the virus, that affected household economic conditions. Being employed at the outbreak of the crisis, facing job interruptions and/or reduction of working hours during the pandemic, increased the risk of financial distress. Interestingly, working from home had, instead, a positive effect. In-line with the literature, education and high levels of income reduced financial distress. We find that the pandemic has worsened economic inequalities, with difficulties that have hit harder households who reported difficulties in the past.

We find that the same variables also play a role in explaining the probability of a worsening in ability to make ends meet between the wave 7 of SHARE (run in 2017–2018) and the SHARE Corona Survey (run in June–September 2020). We observe a prominent role played by age, and income losses during the pandemic and between waves. While being past retirement age has a protective role, income losses increase the probability of financial distress. More in detail, both kinds of losses increase the risk but are the losses during the pandemic to have a larger impact (with a coefficient that is five times that for losses between waves).

This paper provides a new insight on the economic effects of the first wave of the pandemic on 50+ Europeans. It shows that the welfare state effectively protected individuals past retirement age but failed to do the same with younger Europeans (aged 50 or more but below retirement age). Indeed, in this second group the pandemic has hit harder already vulnerable households, thus increasing economic inequality. Our findings could help governments get prepared for future crises and devise more effective policy responses. However, to gain a better understanding of the economic consequences of the pandemic, more data are needed to study not only short-term but also long-term effects.

## Figures and Tables

**Fig. 1:**
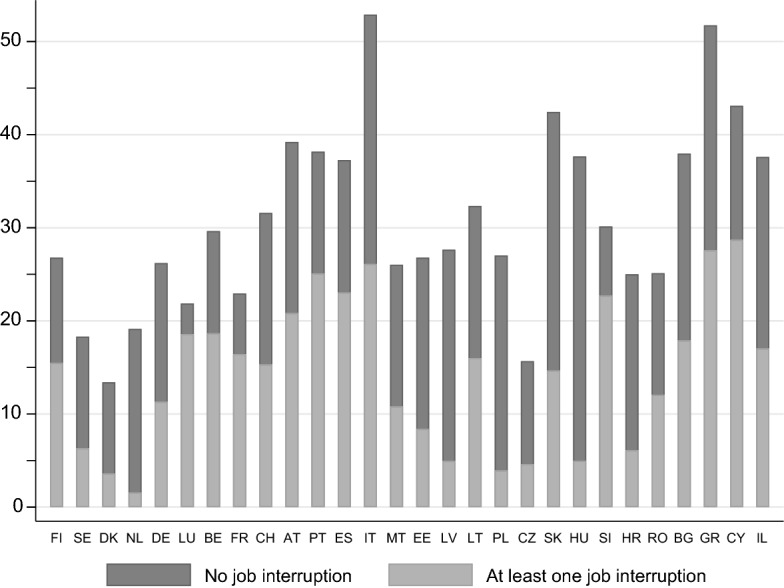
Percentage of households that experienced an income loss and the prevalence of job interruptions among them, by country. The figure shows job interruption occurrence for households that experienced an income loss during the first wave of the pandemic (SCS), using calibrated cross-sectional household weights and multiple imputations. We consider an income loss when the lowest household income during the pandemic is at least 5% lower than the typical household income before COVID-19 broke out. For each country, the figure shows the percentage of employed households that reported an income loss (height of the bars). Each country bar is then split according to job interruption occurrence (i.e. if at least one household respondent who was employed at the outbreak of the pandemic had one or more job interruptions during the pandemic). *Source* Authors’ elaboration using SCS and SHARE waves 1, 2, 4, 5, 6, 7, and 8 data (graphics program: STATA)

**Fig. 2:**
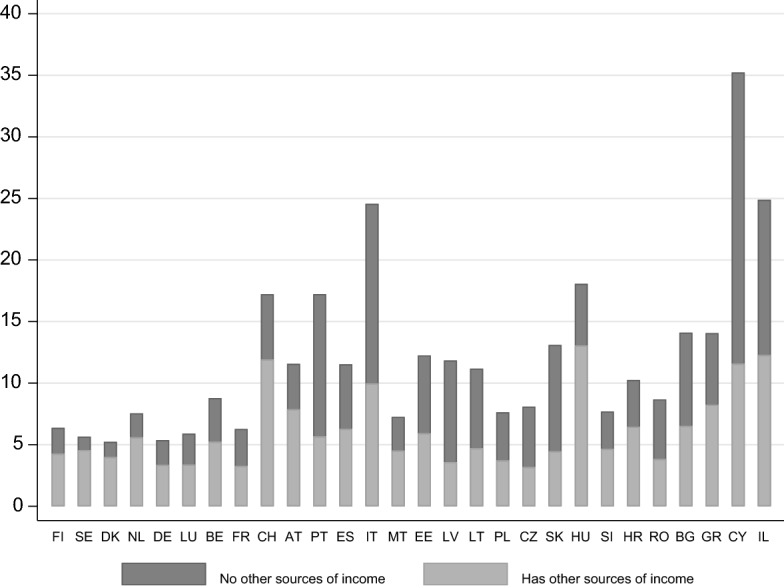
Percentage of households with an income loss but no job interruption/hour reduction and the prevalence of other income sources among them, by country. The figure shows the percentage of households that experienced an income loss but neither job interruption nor hour reduction with respect to other sources of income, during the first wave of the pandemic (SCS), using calibrated cross-sectional household weights and multiple imputations. We consider an income loss when the lowest household income during the pandemic is at least 5% lower than the typical household income before COVID-19 broke out. For each country, the figure shows the percentage of households who reported an income loss (height of the bars). Each country bar is then split according to receiving other sources of income. *Source* Authors’ elaboration using SCS and SHARE waves 1, 2, 4, 5, 6, 7, and 8 data (graphics program: STATA)

**Fig. 3:**
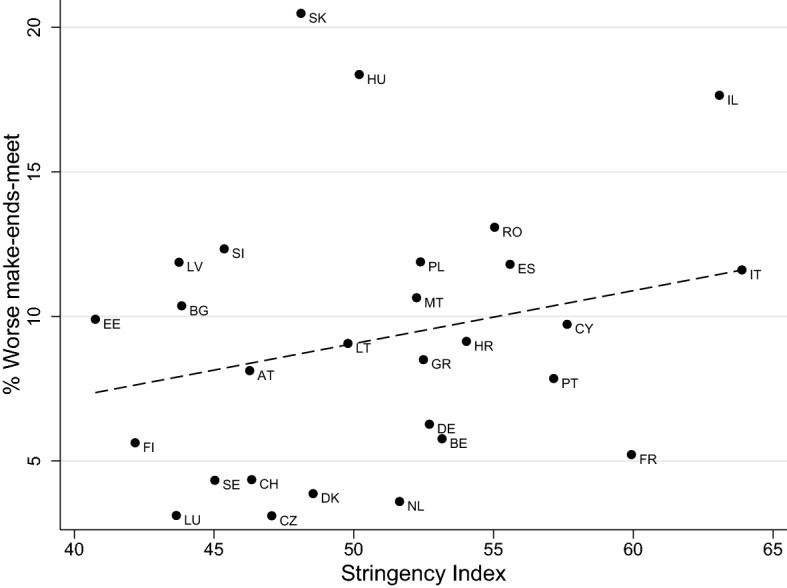
Worsening of ability to make ends meet and intensity of restrictions. The figure shows, for each country, the percentage of households that worsened the economic condition, measured by a worsening in making ends meet (*y*-axis), and the restriction severity (*x*-axis), using calibrated cross-sectional household weights and multiple imputations. Worsening in making ends meet is defined as the transition from no difficulties in making ends meet (SHARE w7) to some or great difficulties (SCS). We compute restriction severity in two steps. We first compute, for each household, the average stringency index for the time window starting from the first week in which restrictions were introduced to the week of the household interview. Then, we take country-level averages of household intensity of restrictions. *Source* Authors’ elaboration using SCS and SHARE waves 1, 2, 4, 5, 6, 7, and 8 data (graphics program: STATA)

## Data Availability

This paper uses data from SHARE Waves 1, 2, 4, 5, 6, 7 and 8 (DOIs: 10.6103/SHARE.w1.710, 10.6103/SHARE.w2.710, 10.6103/SHARE.w4.710, 10.6103/SHARE.w5.710, 10.6103/SHARE.w6.710, 10.6103/SHARE.w7.711, 10.6103/SHARE.w8ca.800, 10.6103/SHARE.w8caintd.800), see Börsch-Supan et al. (2013) for methodological details.

## Appendix

### 6.1

Since Wave 1, SHARE has produced imputations of the missing values due to item non-response by two methods: sequential hot-deck imputations and imputations based on the Fully Conditional Specification (FCS) approach of van Buuren et al. (1999) [also known as Multivariate Imputation using Chained Equations (MICE) or Sequential Regression Multivariate Imputations (SRMI; Raghunathan et al. 2001)].^13^ The first method is used to impute a variety of variables with negligible fractions of missing data (generally much less than 5%), while the second method is typically used to impute financial variables which present considerably smaller response rates. A detailed description of the imputation model can be found in the SHARE Release Guide 8.0.0.^14^

Despite important differences in the underlying data collection process (e.g. CATI versus CAPI interview modes), the non-response patterns of the 1st SHARE Corona Survey (SCS) are quite similar to those observed in the regular waves of SHARE. In particular, there are only two variables with a response rate smaller than 80%: overall monthly household income before the outbreak of COVID-19 (CAHH017) and lowest overall monthly household income since the outbreak of COVID-19 (CAE005). The response rate of many other variables is generally greater than 97%. As in the previous regular waves, variables with negligible fractions of missing data were imputed sequentially by hot-deck, while variables related to hours of work and economic conditions were imputed jointly by the FCS method. Unfortunately, as of July 2022, the available FCS imputations of the variables of interest still suffer from noticeable outliers that may have high leverage on the analysis of the complete data. This is likely due to the fact that FCS imputations of (transformed) financial variables were based on linear regression models. Moreover, unlike the CAPI questionnaires of the regular SHARE waves, the CATI questionnaire of SCS does not include sequences of unfolding bracket questions that may provide valuable interval data on the missing monetary amounts to be imputed.

To overcome this issue, we produced our own FCS imputations for the set of variables that are relevant in our analyses. Following the SHARE practice, we imputed missing data due to “Don’t know” and “Refusal” answers, and, for some variables, other types of data inconsistencies due to routing errors in Section E (Economic situation), measurement errors in Section W (Work), and outliers of the income variables (CAHH017 and CAE005) of the 1st SCS questionnaire.^15^

Specifically, we imputed individual- and household-level variables through a two-step imputation process. In the first step, we imputed jointly a set of eight individual-level variables: years of education, a binary indicator for good self-perceived health before the outbreak of COVID-19 (based on CAPH003), a binary indicator for changes in the self-perceived health status during the outbreak (based on CAH002), employment status at the time when COVID-19 broke out (CAEP805), the occurrence of job interruptions due to the COVID-19 crisis (CAW002), the number of weeks of job interruption (CAW003), the place of work (CAW010), and occurrence of a reduction in working hours since the outbreak of COVID-19 (CAW021). We stress again that individual-level variables present negligible fractions of missing data.

In the second step, we used the complete data (observed plus imputed values) of the variables obtained from the first step to construct the FCS imputations of five household-level variables: overall monthly household income before the outbreak (CAHH017), lowest overall monthly household income since the outbreak (CAE005), household’s ability to make ends meet (CACO007), a binary indicator for the postponement of regular payments (CAE011) and a binary indicator for dipping into savings to cover the necessary day-to-day expenses (CAE012).

FCS imputations of these thirteen variables were always constructed separately by country. At each iteration of the FCS algorithm (STATA command mi impute chained), we used a predictive mean matching model (STATA command mi impute pmm) for the continuous variables (years of education, CAW003, CAHH017, and CAE005),^16^ a logit model (STATA command mi impute logit) for seven binary variables (good self-perceived health before the outbreak of COVID-19, changes in the self-perceived health status during the outbreak, CAEP805, CAW002, CAW021, CAE011 and CAE012), an ordered logit model (STATA command mi impute ologit) for the categorical variable CACO007, and a multinomial logit model (STATA command mi impute mlogit) for the categorical variable CAW010.

In addition to the variables jointly imputed in the FCS method, in the first stage, we used as observed predictors a binary indicator for female respondents, age, and a binary indicator for living with a spouse/partner. This holds for all imputed variables except for years of education and the two health-related variables. To impute these three variables, we used only the three observed predictors (gender, age, and couple). In the second stage, instead, we used as observed predictors, the imputed variables from the first stage for the first member of the household, a binary indicator for gender, age, and years of education of the first respondent, a binary indicator for living with a spouse/partner, and its interactions with the imputed variables from the first stage for the second member of the household, age, and years of education of the spouse/partner.

Lastly, we refined the choice of the predictors in the final FCS imputation model adopted in each country to avoid possible problems of collinearity, convergence, and perfect prediction. To this end, we (parsimoniously) imposed a set of country and item-specific exclusion restrictions. Note that in our setting we end up jointly imputing missing values on variables that are logically related. After an initial set of 10 burn-in iterations, convergence was achieved before the 50th iteration in all countries.

To account for the additional variability induced by the imputation process, we run five independent replicas of the FCS imputation method thus obtaining five different imputations of the missing values.

Stata programs for our multiple FCS imputations are available from the authors upon request.

Table 9 compares the distributions of original and complete data (imputed and observed) in terms of mean and standard error. Columns 4, and 5 present the moments over the complete data obtained using our imputations, while columns 6 and 7 show results using SHARE imputations (public-use data). All individual-level variables present low fractions of imputed data, usually well below 5%, and complete data moments, for both ours and SHARE imputations, in-line with the original data counterparts. At the household level, instead, all variables required imputations for more than 3.5% of the data, with a peak of almost 27% for the two income variables (“Income before COVID-19” and “Lowest income during COVID-19”). For all household-level variables, except the income variables, means and standard errors show similar results in all three specifications.

**Table 9:** Description of imputed variables

	Imputation method—authors	Original data		Complete data (Imputed + Observed data)—Authors		Complete data (Imputed + Observed data)—Public-use		Imputed data
		Mean	SE	Mean	SE	Mean	SE	
*Individual-level variables*								
Years of education	pmm (knn = 10)	11.10	4.22	11.09	4.23	11.09	4.23	3.74%
Changed health status	Logit	12.00%	32.50%	12.00%	32.50%	12.00%	32.50%	0.09%
Good health status	Logit	66.99%	47.02%	66.98%	47.03%	66.98%	47.03%	0.09%
Employed	Logit	20.78%	40.58%	20.78%	40.57%	20.78%	40.58%	0.16%
Job interruption	Logit	19.07%	39.28%	19.05%	39.27%	19.09%	39.30%	0.16%
Weeks of job interruption	pmm (knn = 1)	8.73	4.68	8.70	4.69	8.75	4.68	4.09%
Place of work:	mlogit	2.31	0.94	2.31	0.94	2.31	0.94	0.28%
Home only		16.62%		16.62%		16.62%		
Usual workplace		52.31%		52.32%		52.29%		
Home and Usual workplace		14.09%		14.09%		14.11%		
None of these		16.98%		16.97%		16.98%		
Reduced working hours	logit	20.76%	40.56%	20.97%	40.71%	20.89%	40.65%	3.05%
*Household-level variables*								
Income before COVID-19	pmm (knn = 10)	1682.52	1436.14	1740.54	1682.24	1739.66	1531.92	26.91%
Lowest income during COVID-19	pmm (knn = 10)	1622.73	1415.29	1504.77	1476.36	1919.80	27,668.54	26.68%
Ability to make-ends-meet	Ologit	2.85	0.96	2.84	0.96	2.84	0.96	3.60%
With great difficulty		9.65%		9.68%		9.68%		
With some difficulty		25.82%		25.94%		25.95%		
Fairly easily		34.84%		34.83%		34.85%		
Easily		29.68%		29.55%		29.52%		
Postponed payments	Logit	9.57%	29.41%	9.53%	29.39%	9.56%	29.40%	4.24%
Dip into savings	Logit	18.27%	38.64%	18.32%	38.68%	18.29%	38.66%	4.55%

The table reports imputation method, mean, standard error of the mean, and percentage of imputed values of our variables of interest. Column 1 shows the imputation method used to produce our imputations, columns 2 and 3 show the results for the original data, columns 4 and 5 report mean and standard error for the complete data using our imputations, columns 6 and 7 the moments for the public-use complete data (using SHARE imputations), while column 8 shows the percentage of imputed data. Our variables of interest include: years of education (Yedu), a binary indicator for good self-perceived health before the outbreak of COVID-19 (d_sphus, based on CAPH003), a binary indicator for changes in the self-perceived health status during the outbreak (d_csphus, based on CAH002), employment status at the time when COVID-19 broke out (empself, CAEP805), a binary indicator of occurrence of job interruptions due to the COVID-19 crisis (unemployed, CAW002), the number of weeks of job interruption (wunemployed, CAW003), the place of work (pwork, CAW010), a binary indicator of occurrence of a reduction in working hours since the outbreak of COVID-19 (rwhours, CAW021), the overall monthly household income before the outbreak of COVID-19 (mthinc2, CAHH017), the lowest overall monthly household income since the outbreak (lmthinc2, CAE005), household’s ability to make ends meet (cahmem, CACO007), a binary indicator for the postponement of regular payments (prbills, CAE011), and a binary indicator for dipping into savings to cover the necessary day-to-day expenses (dipsav, CAE012)

*Source* Authors’ elaboration using SCS and SHARE waves 1, 2, 4, 5, 6, 7, and 8 data

As for the income variables, complete data moments differ only slightly from the original data using our imputations. The same is true for the “Income before COVID-19” variable in the SHARE imputations, but not for the “Lowest income during COVID-19”. For this variable both mean and standard error are much larger than in the original data.

Table 10 shows the percentage of households that report an “Income before COVID-19” larger, equal, or smaller than the “Lowest income during COVID-19”. The results in Table 10 are similar among specifications and do not stand against the use of public-use data. Despite this, focusing on the entire income distributions, Table 11 shows the presence of outliers in the public-use data. Maximum values, for both the “Income before COVID-19” and the “Lowest income during COVID-19” equal 12,000€ in the original data as well as in the complete data using our imputations. In the public-use data, instead, maximum values are, respectively, 53,478.66€ and 11,654,756.53€.

**Table 10:** Imputed income variables

	Original data	Authors imputations	Public-use data
Income before COVID-19 > Lowest income during COVID-19	43.25%	46.50%	47.52%
Income before COVID-19 = Lowest income during COVID-19	6.11%	5.27%	4.36%
Income before COVID-19 < Lowest income during COVID-19	50.64%	48.23%	48.12%
	100%	100%	100%
Obs	27,931	195,520	195,520

The table shows the percentage of households that report an “Income before COVID-19” larger, equal, or lower than the “Lowest income during COVID-19” in the original data (column 1) and in the complete data using our imputations (column 2) and SHARE imputations (column 3)

*Source* Authors’ elaboration using SCS data

**Table 11:** Income variables distribution

	Original data		Complete data (imputed + observed data)—authors		Complete data (imputed + observed data)—public-use	
	Income before COVID-19	Lowest income during COVID-19	Income before COVID-19	Lowest income during COVID-19	Income before COVID-19	Lowest income during COVID-19
Mean	1682.52	1622.73	1740.54	1682.24	1739.66	1919.80
SE	1436.16	1415.31	1504.77	1476.36	1531.92	27,668.54
Min	82.67	0.00	82.67	0.00	26.64	0.00
Max	12,000.00	12,000.00	12,000.00	12,000.00	53,478.66	11,654,765.53
p1	172.45	122.71	178.95	119.39	172.45	75.00
p5	320.00	284.70	332.34	298.93	320.39	270.09
p10	430.00	400.00	450.00	410.00	436.87	400.00
p25	676.62	650.00	700.00	675.22	700.00	650.00
p50	1200.00	1200.00	1235.00	1200.00	1240.00	1200.00
p75	2200.00	2100.00	2300.00	2200.00	2300.00	2200.00
p90	3500.00	3500.00	3800.00	3600.00	3736.57	3756.27
p95	4670.71	4500.00	4829.49	4670.71	4800.00	5000.00
p99	6819.24	6707.63	7378.39	7006.07	7401.23	8407.29
Obs	28,582	28,673	195,520	195,520	195,520	195,520

The table shows descriptive statistics (mean, standard error of the mean, minimum, maximum, 1st percentile, 5th percentile, 10th percentile, 25th percentile, median, 75th percentile, 90th percentile, 95th percentile, 99th percentile, and number of observations) of the “Income before COVID-19” and of the “Lowest income during COVID-19”. Columns 1 and 2 show the results for the original data, columns 3 and 4 report the descriptive statistics for the complete data using our imputations, and columns 5 and 6 the results for the public-use complete data (using SHARE imputations)

*Source* Authors’ elaboration using SCS data

### 6.2

In this subsection, we present descriptive analyses of economic outcomes (income losses, and variation in ability to make ends meet) during the first wave of the pandemic for different subsamples of the population of interest. We first investigate the relation between employment and income losses, then we move to education and ability to make ends meet.

#### Employment and economic outcomes

Figure 1 shows the incidence of job interruptions among employed households with an income loss. We consider an income loss when the lowest household income during the pandemic is at least 5% lower than the typical household income before COVID-19 broke out. We choose the 5% threshold to account for possible errors and/or rounding in reporting the amounts.

As expected, Fig. 1 shows a link between job interruptions and income loss. In the whole subsample of employed households with an income loss, 48.18% also experienced at least one job interruption. This is true for more than 50% of households in thirteen countries (Finland, Luxembourg, Belgium, France, Switzerland, Austria, Portugal, Spain, Italy, Lithuania, Slovenia, Greece, and Cyprus).

Figure 1 shows that among employed households with income loss 51.82% did not report any job interruption. Table 12 focuses on employed households who experienced an income loss but no job interruption.^17^ It displays how many of these households reduced their working hours during the first wave of the pandemic, i.e. if at least one household member who was employed when COVID-19 broke out reduced working hours during the pandemic.

**Table 12:** Percentage of employed households with a reduction in working hours given an income loss but no job interruption, by country

Country	At least one reduction (%)	No reduction (%)	Obs	Country	At least one reduction (%)	No reduction (%)	Obs
Austria	30.13	69.87	30.4	Hungary	66.99	33.01	17.8
Germany	64.70	35.3	69.2	Portugal	53.53	46.47	14.0
Sweden	41.78	58.22	31.2	Slovenia	56.28	43.72	17.2
Netherlands	61.69	38.31	11.2	Estonia	39.62	60.38	163.0
Spain	57.12	42.88	14.6	Croatia	22.58	77.42	31.0
Italy	31.39	68.61	103.0	Lithuania	29.74	70.26	43.6
France	21.51	78.49	17.4	Bulgaria	14.15	85.85	21.6
Denmark	35.68	64.32	46.0	Cyprus	0	100	3.2
Greece	23.83	76.17	58.8	Finland	57.87	42.13	21.8
Switzerland	53.12	46.88	69.6	Latvia	17.25	82.75	26.4
Belgium	25.87	74.13	75.8	Malta	40.72	59.28	9.4
Israel	30.52	69.48	51.6	Romania	36.91	63.09	17.6
Czech Rep	32.05	67.95	59.8	Slovakia	34.93	65.07	54.4
Poland	42.42	57.58	75.4				
Luxembourg	23.60	76.4	3.8	Total	44.49	55.51	1158.8

The table shows, for employed households that experienced an income loss but did not incur in job interruptions during the first wave of the pandemic (SCS), the percentage of households that reported a reduction in working hours, using calibrated cross-sectional household weights and multiple imputations. We consider an income loss when the lowest household income during the pandemic is at least 5% lower than the typical household income before COVID-19 broke out. We account for a working hours reduction occurrence when at least one household respondent who was employed at the outbreak of the pandemic reduced working hours during the pandemic. Since the variables job interruptions and reduction in working hours are collected conditional on being in employment, imputations are computed only when the condition is met, and the average number of observations can be not an integer

*Source* Authors’ elaboration using SCS and SHARE waves 1, 2, 4, 5, 6, 7, and 8 data.

Among employed households with income loss and no job interruption, 44.49% of them reported a reduction of working hours, but with marked country differences. Alternative possible explanations for income loss (in case of no job interruption and no reduction of working hours) lie in the reduction of other sources of income, such as income from household members other than the respondents (such as co-resident grown children), “second homes” (that is housing stock that is not used as main residence), financial investments, and businesses.

Table 13 shows the distribution of these other sources of income in our sample. More than half of the sample (50.38% of households) has at least another source of income in addition to employment and/or pension incomes of members included in the interview. More in detail, 21.39% of households can count on income flows from other household members, 23.06% own “second homes”, 22.57% hold financial assets, and 9.77% are business owners (either entirely or partially). We can also see from Table 13 that 16.41% of respondents are tenants or subtenants of the dwelling they live in, while the remaining 83.59% are homeowners, rent-free, or members of a cooperative.

**Table 13:** Summary statistics (waves 1–2, 4–8)—other sources of income and homeownership

Outcomes (*Household level*)	Mean (%)	SE (%)	Obs
Other sources of income (at least one)	50.38	0.28	31,227
Income from other household members	21.39	0.23	31,227
Owners of second homes	23.06	0.24	31,227
Financial assets (stocks, bonds, mutual funds)	22.57	0.24	31,227
Business owners	9.77	0.17	31,227
Tenant/subtenant	16.41	0.21	31,227

The table shows descriptive statistics (mean, standard error of the mean, and number of observations), using calibrated cross-sectional household weights and multiple imputations, of other sources of income and home ownership (most recent information collected in waves 1, 2, 4, 5, 6, 7, and 8). Other sources of income is a dummy indicator that takes value 1 if the household reports having at least one of the following income sources: Income from other household members not included in the interview, second homes, investments in financial assets, and businesses. Tenant is a dummy variable that equals 1 if the household occupies the dwelling they live as tenant or subtenant

*Source* Authors’ elaboration using SCS and SHARE waves 1, 2, 4, 5, 6, 7, and 8 data

Information in Table 13 can help explain the drivers of income losses households experienced during the pandemic. Thus, we move here our focus from employed households, as in Fig. 1 and Table 12, to all households. We can see from Fig. 2 that a large fraction of households without both hour reduction and job interruption but with income loss, have additional sources of income. Among households without hour reduction and job interruption only about 11% reported an income loss. Pooling all countries together, we observe that most households have other sources of income (average percentage: 50.80%). At the country level, instead, we observe large percentages for Nordic countries and low percentages for Eastern countries.

#### The role of education and age

Table 14 suggests a protective role of education on the variation in ability to make ends meet between SHARE w7 and the SCS. Table 14 shows that the fraction of households who reported any variation in ability to make ends meet decreases as education increases (even if not monotonically). Similarly, the percentage of households who worsened their economic condition, moving from no difficulties to some or great difficulties, decreases (from 10.68% to 6.50%) with education.

**Table 14:** Percentage change in ability to make ends meet between wave 7 and SCS by education

	Primary (or none)	Low secondary	Upper secondary	Post-secondary	Tertiary
No variation	70.18	71.34	75.95	82.00	82.80
Worsening	10.68	9.56	9.33	6.21	6.50
Improvement	19.14	19.09	14.72	11.80	10.70
Observations	2944	4420	13,411	3887	6565

The table shows household variation in ability to make ends meet between wave 7 (2017) and the SCS (first wave of the pandemic—2020) by household level of education (i.e. the level of education of either the respondent—single household—or the most educated couple respondent), using calibrated cross-sectional household weights and multiple imputations. For each level of education, the table shows the percentage of households that had some or great difficulties in making ends meet in wave 7 but that did not have those difficulties during the first wave of the pandemic (Improvement), the percentage of households that have experienced a worsening of their ability to make ends meet (from no difficulties to some or great difficulties—Worsening), and the percentage of households that did not experience any variation between the two surveys (either they did not have or they had difficulties in making ends meet in both surveys—No variation)

*Source* Authors’ elaboration using SCS and SHARE waves 1, 2, 4, 5, 6, 7, and 8 data

### 6.3

In this subsection, we present our estimation results when the dependent variable is a measure of COVID-19 impact on household economic conditions. Table 15 reports the OLS estimates for the models that explain difficulty in making ends meet, postponed payments, dipped into savings, and income loss.

**Table 15:** OLS regressions—dependent variables: difficulties in MeM, postponed payments, dip into savings, and income loss

	MeM_SCS_	Postpay	DipSav	IncLoss_scs_
Female	− 0.003	− 0.008	− 0.006	− 0.012*
	(0.006)	(0.009)	(0.012)	(0.007)
Household size	0.021***	0.012***	0.003	0.013***
	(0.003)	(0.003)	(0.004)	(0.003)
*HH composition and age*				
Single ≥ 65	− 0.082***	− 0.059***	− 0.029**	− 0.034***
	(0.009)	(0.012)	(0.014)	(0.009)
Couple < 65	0.020*	− 0.001	− 0.007	− 0.048***
	(0.010)	(0.013)	(0.017)	(0.012)
Couple ≥ 65	− 0.035***	− 0.050***	− 0.053***	− 0.070***
	(0.010)	(0.013)	(0.016)	(0.010)
Years of education	− 0.004***	0.001	0.007***	0.000
	(0.001)	(0.001)	(0.001)	(0.001)
Employed	− 0.006	0.004	0.018	0.076***
	(0.008)	(0.011)	(0.015)	(0.009)
Job interruption	− 0.016	0.131***	0.205***	0.213***
	(0.021)	(0.037)	(0.046)	(0.028)
Home working	− 0.016*	0.035*	− 0.019	− 0.051***
	(0.009)	(0.020)	(0.025)	(0.010)
Reduced working hours	0.024**	0.025	0.049*	0.204***
	(0.011)	(0.021)	(0.028)	(0.015)
Weeks of job interruption	0.013***	0.002	0.005	0.017***
	(0.002)	(0.003)	(0.004)	(0.003)
Income from others	0.009*	0.003	− 0.001	0.007
	(0.006)	(0.007)	(0.010)	(0.005)
Second homes	− 0.011**	− 0.005	0.009	− 0.001
	(0.005)	(0.008)	(0.010)	(0.005)
Investments	− 0.033***	− 0.020*	− 0.006	− 0.008
	(0.005)	(0.012)	(0.019)	(0.006)
Own business	0.039***	0.051***	0.085***	0.061***
	(0.009)	(0.017)	(0.022)	(0.010)
Tenant	0.049***	0.049***	− 0.001	0.005
	(0.007)	(0.010)	(0.013)	(0.006)
log(Income before COVID)	− 0.225***	− 0.056***	− 0.007	0.024***
	(0.006)	(0.008)	(0.010)	(0.005)
Length of restrictions	0.002	− 0.006***	− 0.003	− 0.000
	(0.001)	(0.002)	(0.002)	(0.001)
Avg. restriction intensity	− 0.001	− 0.007	0.000	0.000
	(0.004)	(0.005)	(0.008)	(0.004)
MeM wave7	0.246***	0.027***	0.013	0.013***
	(0.006)	(0.006)	(0.009)	(0.005)
Observations	31,227	10,537–10,575	10,537–10,575	31,227
*R*^2^	0.397	0.080	0.105	0.175

Dependent variables are binary indicators for: (some or great) difficulties in making ends meet during the pandemic (column 1), postpone payment (column 2), dip into savings (column 3), and income losses during the pandemic (column 4). The results obtained using multiple imputation technique for missing values. Note that since the variables postpone payment (Postpay) and dip into savings (DipSav) are collected conditional on reporting some or great difficulties in making ends meet, imputations are computed only when the condition is met, and estimation subsample vary across imputations in columns 2 and 3. Robust standard errors in parentheses. ****p * < 0.01; ***p* < 0.05; **p* < 0.1

*Source* Authors’ elaboration using SCS and SHARE waves 1, 2, 4, 5, 6, 7, and 8 data

Columns 1, and 4 present estimates over the full sample, while columns 2 and 3 show results for the subsample of households who reported difficulties in making ends meet. In all specifications, we use the following controls at the household level: country dummies, gender, household size, household type (i.e. single vs. couple) by age (young olds vs. old olds), years of education, employment status, occurrence of job interruptions, working from home, reduction of working hours, number of weeks of job interruption, other sources of income (i.e. income from other household member, second homes, investments, and businesses), being tenant/subtenant, income before the COVID-19 crisis, length and intensity of restrictions, ability to make ends meet reported in wave 7. See footnote 11 for more details.

The results in columns 1 show only few differences with respect to column 2 of Table 7 that are worth underlying. First, being in a couple under 65 increases the probability to report difficulties in making ends meet (positive and significant coefficient for “couple < 65”). Second, among employment variables, only “Home working” (negative and significant coefficient), “Reduced working hours”, and “Weeks of job interruption” are relevant (positive and significant coefficients). Lastly, owning “Second homes” reduces the probability of reporting make ends meet difficulties.

Columns 2 and 3 show the OLS estimates using as dependent variable a dummy for either postponed payments or dipped into savings. In both regressions, age plays a protective role for singles and couples with negative and significant coefficients for “single ≥ 65” and “couple ≥ 65”. “Job interruption” increases the probability to postpone payments and dip into savings, while “Reduced working hours” shows a positive and significant coefficient only in column 2. “Own business” has a significant and negative effect (positive coefficients) for both the probability to postpone payments and to dip into savings, while being “Tenant” and having “Investments”, respectively, increases and decreases the probability to postpone payments. The coefficient of “log(Income before COVID-19)” is negative but significant only in the postponed payment regression (column 2). As regard government policies, the results show that only the “Length of restrictions” is significant, with a negative sign, in column 2. While “Years of education” is not relevant for ”Postpay”, its coefficient is significant and positive for “DipSav”, indicating that education increases the probability to resort to accumulated savings in case of need. Note, however, that results in column 3 may be misleading as failing to use savings may be due to two different kinds of reasons: no need to use savings and having no savings to begin with. Consequently, results for education may reflect more the availability than the need to use savings. “Make-ends-meet wave 7” is positive and significant in column 2, confirming that the pandemic has exacerbated economic inequalities.

We can see in column 4 how our regressors affect the probability to suffer from an income loss during the first wave of the pandemic. Income loss is defined as a dummy variable that equals one when the lowest household income during the pandemic is at least 5% lower than the typical household income before the outbreak. “Household composition and age” plays an important role, with all coefficients that are negative and significant. Thus, being 65 or more and/or having a partner reduces the probability to suffer from an income loss. All employment variables (“Employed”, “Job interruption”, “Home working”, “Reduced working hours”, and “Interruption weeks”), but “Home working”, have a negative and significant effect (positive coefficients). Coefficient for “Home working”, instead, is negative and significant. “log(Income before COVID-19)” increases the probability of an income loss (positive and significant coefficient). Among the other possible sources of income, only the “Own business” coefficient is significant with a positive sign. Lastly, “MeM wave7” negatively affect the probability with a positive and significant coefficient.

Table 16 investigates which factors affect the probability to experience an income loss during the pandemic, distinguishing between all households (column 1), households with a job interruption or a reduction of working hours (column 2), and households without interruptions and reductions (column 3). Note that column (1) is the same regression as in column 4 of Table 15. The two subsamples on which we focus in columns 2 and 3 are justified by the prominent role that job interruption and hour reduction show in column 1. The results in column 3 are in-line with column 1 and suggest that these households (that represent about 92% of the sample) drive the results for the full sample. Columns 4 and 5 focus on the same subsamples of columns 2 and 3 but use as dependent variables income variations, in percentage terms, during the pandemic (they span from negative to positive values).

**Table 16:** OLS regressions—Dependent variables: income loss during the pandemic, percentage income variation during the pandemic

	Income loss (SCS)			% Income change (SCS)	
	(full sample)	JI or RWH	No JI and No RWH	JI or RWH	No JI and No RWH
Female	− 0.012*	− 0.047	− 0.011	− 15.770	0.038
	(0.007)	(0.034)	(0.007)	(15.680)	(0.583)
Household size	0.013***	0.027**	0.013***	1.088	0.215
	(0.003)	(0.012)	(0.003)	(2.189)	(0.290)
*HH composition and age:*					
single ≥ 65	− 0.034***	0.025	− 0.038***	− 0.817	2.080**
	(0.009)	(0.040)	(0.010)	(9.998)	(0.846)
Couple < 65	− 0.048***	− 0.089***	− 0.042***	25.541*	6.005***
	(0.012)	(0.035)	(0.013)	(13.559)	(1.833)
Couple ≥ 65	− 0.070***	− 0.104**	− 0.070***	21.981**	6.626***
	(0.010)	(0.043)	(0.011)	(9.462)	(1.810)
Years of education	0.000	0.001	0.000	1.687	0.074
	(0.001)	(0.004)	(0.001)	(1.472)	(0.086)
Employed	0.076***		0.051***		1.478
	(0.009)		(0.009)		(1.182)
Job interruption	0.213***				
	(0.028)				
Home working	− 0.051***	− 0.089***	− 0.017	13.512	1.761**
	(0.010)	(0.024)	(0.011)	(9.874)	(0.865)
Reduced working hours	0.204***				
	(0.015)				
Weeks of job interruption	0.017***	0.016***		− 1.256***	
	(0.003)	(0.002)		(0.335)	
Income from others	0.007	− 0.015	0.011**	− 3.642	− 0.309
	(0.005)	(0.026)	(0.005)	(3.802)	(0.602)
Second homes	− 0.001	0.001	0.001	− 2.934	− 0.162
	(0.005)	(0.023)	(0.005)	(2.480)	(0.508)
Investments	− 0.008	− 0.026	− 0.005	9.689	0.436
	(0.006)	(0.027)	(0.007)	(10.577)	(0.626)
Own business	0.061***	0.116***	0.045***	1.017	− 2.402***
	(0.010)	(0.026)	(0.010)	(7.906)	(0.771)
Tenant	0.005	0.036	0.001	11.561	− 1.270**
	(0.006)	(0.031)	(0.006)	(13.957)	(0.553)
log(Income before COVID)	0.024***	0.053**	0.020***	− 56.671	− 10.465***
	(0.005)	(0.025)	(0.005)	(42.565)	(3.424)
Length of restrictions	− 0.000	0.003	− 0.000	0.251	0.190
	(0.001)	(0.006)	(0.001)	(0.768)	(0.199)
Avg. restriction intensity	0.000	− 0.005	0.001	− 6.114	0.849
	(0.004)	(0.023)	(0.004)	(4.844)	(0.611)
MeM wave7	0.013***	0.038*	0.011**	− 14.290	− 2.431***
	(0.005)	(0.023)	(0.004)	(10.338)	(0.777)
Observations	31,227	2567–2581	28,646–28,660	2567–2581	28,646–28,660
*R*^2^	0.175	0.115	0.050	0.082	0.021

Dependent variables in columns 1–3 are binary indicators for income losses during the pandemic. Column 1 refers to the entire sample. Column 2 focuses on the subsample of households that reported either a “Job interruption” (JI) or “Reduced Working Hours” (RWH). Column 3 refers to the subsample of households that did not have “Job interruption” (JI) or “Reduced Working Hours” (RWH). Columns 4 and 5 focus on the same subsamples of columns 2 and 3 but use as dependent variables income variations, in percentage terms, during the pandemic (they span from negative to positive values). The results obtained using multiple imputation technique for missing values. Note that since the variables job interruption and reduction in working hours are collected conditional on being employed, imputations are computed only when the condition is met, and estimation subsample vary across imputations in columns 2–5. Robust standard errors in parentheses. ****p * < 0.01; ***p* < 0.05; **p* < 0.1

*Source* Authors’ elaboration using SCS and SHARE waves 1, 2, 4, 5, 6, 7, and 8 data

Finally, Table 17 reports the OLS estimates for the models that explain make ends meet worsening (columns 1–3) and improvement (columns 4–6). See footnote 12 for details on regression variables. The results in columns 1 and 4 are presented also in Table 8. As previously discussed, column 1 shows a protective role of age, income and education, and a detrimental role of income losses. Among employment variables, instead, the number of “Weeks of job interruption” during the first wave of the pandemic significantly increases the probability of a worsening. Moving to other sources of income, “Second homes” and “Investments” reduce the probability of a deterioration in ability to make ends meet, while the coefficient for “Own business” is positive and significant. Lastly, being “Tenant” and the “Length of restrictions” negatively affect make ends meet variation. Estimation results for “make ends meet” improvements, column 4, mirror, in general, the results in column 1.

**Table 17:** OLS—Dependent variables: worsening and improvement in making ends meet

	MeM worsening			MeM improvement		
		(Income loss)	(No income loss)		(Income gain)	(No income gain)
Female	− 0.009	− 0.016	− 0.001	− 0.017	− 0.016	− 0.017
	(0.007)	(0.012)	(0.008)	(0.012)	(0.017)	(0.018)
Household size	0.016***	0.007	0.022***	− 0.022***	− 0.032***	− 0.016***
	(0.004)	(0.006)	(0.006)	(0.005)	(0.007)	(0.006)
plusDeltaSize	− 0.004	0.011	− 0.016	− 0.008	− 0.022	0.022
	(0.023)	(0.036)	(0.030)	(0.028)	(0.038)	(0.042)
minDeltaSize	0.021**	0.015	0.013	0.010	− 0.009	0.016
	(0.009)	(0.012)	(0.013)	(0.013)	(0.023)	(0.018)
*HH composition and age:*						
Single ≥ 65	− 0.074***	− 0.093***	− 0.040**	0.075***	0.071***	0.085***
	(0.012)	(0.019)	(0.016)	(0.014)	(0.020)	(0.021)
Couple < 65	0.000	0.003	0.009	− 0.044***	− 0.056**	− 0.024
	(0.013)	(0.020)	(0.017)	(0.017)	(0.024)	(0.025)
Couple ≥ 65	− 0.038***	− 0.049**	− 0.013	0.023	0.014	0.038
	(0.013)	(0.020)	(0.017)	(0.016)	(0.024)	(0.025)
Years of education	− 0.004***	− 0.005***	− 0.002**	0.003**	0.003	0.003*
	(0.001)	(0.001)	(0.001)	(0.001)	(0.002)	(0.002)
*Employment variation:*						
Always employed	− 0.006	− 0.017	0.009	0.023	0.034	− 0.002
	(0.011)	(0.017)	(0.013)	(0.017)	(0.024)	(0.027)
Employed-Not empl	0.013	0.011	0.006	− 0.012	− 0.020	− 0.020
	(0.010)	(0.014)	(0.014)	(0.017)	(0.029)	(0.021)
Not empl—Employed	0.026*	0.030	0.022	0.022	0.023	0.024
	(0.015)	(0.025)	(0.018)	(0.025)	(0.031)	(0.047)
Job interruption	− 0.048*	− 0.031	− 0.074**	− 0.035	− 0.041	− 0.009
	(0.026)	(0.035)	(0.033)	(0.040)	(0.056)	(0.059)
Home working	− 0.014	− 0.011	− 0.017	0.060***	0.038	0.094**
	(0.009)	(0.015)	(0.010)	(0.023)	(0.029)	(0.038)
Reduced working hours	0.003	− 0.003	0.023	− 0.032	− 0.016	− 0.052
	(0.012)	(0.018)	(0.015)	(0.024)	(0.032)	(0.038)
Weeks of job interruption	0.011***	0.010***	0.010**	− 0.012***	− 0.013**	− 0.010*
	(0.003)	(0.003)	(0.005)	(0.004)	(0.005)	(0.006)
IncLoss_SCS	0.095***	0.078***				− 0.004
	(0.011)	(0.018)				(0.018)
IncLoss_waves	0.019***	− 0.013				0.031**
	(0.006)	(0.021)				(0.013)
IncGain_SCS			0.029	0.049**	0.050	
			(0.025)	(0.019)	(0.030)	
IncGain_waves			− 0.002	0.021**	− 0.006	
			(0.007)	(0.009)	(0.029)	
log(Income before COVID)	− 0.171***	− 0.194***	− 0.146***	0.274***	0.302***	0.252***
	(0.008)	(0.012)	(0.009)	(0.011)	(0.017)	(0.015)
Income from others	0.008	0.010	0.005	− 0.011	− 0.013	− 0.014
	(0.006)	(0.010)	(0.008)	(0.010)	(0.014)	(0.016)
Second homes	− 0.014**	− 0.012	− 0.012*	0.012	0.007	0.017
	(0.006)	(0.009)	(0.007)	(0.010)	(0.015)	(0.015)
Investments	− 0.023***	− 0.037***	− 0.011**	0.093***	0.089***	0.098***
	(0.005)	(0.008)	(0.006)	(0.017)	(0.023)	(0.027)
Own business	0.036***	0.052***	0.017	− 0.052***	− 0.052*	− 0.057**
	(0.009)	(0.014)	(0.011)	(0.020)	(0.028)	(0.029)
Tenant	0.032***	0.047***	0.019**	− 0.080***	− 0.100***	− 0.047**
	(0.008)	(0.013)	(0.009)	(0.012)	(0.017)	(0.019)
Length of restrictions	0.003*	0.003	0.002	− 0.001	0.002	− 0.005
	(0.002)	(0.002)	(0.002)	(0.003)	(0.004)	(0.004)
Avg. restriction intensity	0.001	0.005	− 0.005	0.005	0.008	− 0.001
	(0.004)	(0.007)	(0.006)	(0.008)	(0.012)	(0.013)
Observations	18,337	8815–8858	9479–9522	12,890	7322–7378	5512–5568
R^2^	0.199	0.215	0.146	0.237	0.220	0.233

Dependent variables in columns 1, 2 and 3 are binary indicators for worsening in making ends meet from wave 7 to SCS, for the subsample of households that did not report some or great difficulties in making ends meet in wave7. Dependent variables in Columns 4, 5 and 6, instead, are binary indicators for improvement in making ends meet from wave 7 to SCS, for the subsample of households who had some or great difficulties in wave7. Columns 1 and 4 refer to the full (respective) subsamples. Columns 2 and 5 focus, respectively, on the subsample of households with an income loss or an income gain (either between waves or during the pandemic), while columns 3 and 6 focuses, respectively, on the subsample of households without any income loss or income gain. The results obtained using multiple imputation technique for missing values. Note that since the variables income loss and income gains depend on income values either reported by the respondents or imputed, estimation samples vary across imputations in columns 2–3 and 5–6. Robust standard errors in parentheses. ****p * < 0.01; ***p* < 0.05; **p* < 0.1

*Source* Authors’ elaboration using SCS and SHARE waves 1, 2, 4, 5, 6, 7, and 8 data

We add here four additional regressions that make use of specific subsamples. Columns 2 and 3 show results for a worsening in ability to make ends meet between wave 7 and the SCS, for the subsample of households that did not report some or great difficulties in making ends meet in wave7. Columns 5 and 6, instead, present specular information: improvements in making ends meet for households who had some or great difficulties in wave7. Columns 2 and 5 focus, respectively, on the subsample of households with an income loss or an income gain (either between waves or during the pandemic), while columns 3 and 6 concentrates, respectively, on the subsamples of households without any income loss or income gain.

The results in columns 2 and 3 are like those in column 1 but with some relevant differences. For households who experienced at least an income loss (column 2), coefficients for income losses are positive but significant only for pandemic variations (“IncLoss_SCS”).

Moving to ability improvements in columns 4–6, we can see that many of the results are in-line with columns 1–3, although specular. We can however underline two notable differences. First, not only the coefficient for “single ≥ 65” is positive and significant (in columns 4–6) but also the coefficient for “couple < 65” is negative and significant in columns 4 and 5, meaning that having a partner reduces the probability of an improvement. Second, “Home working” increases the probability of an ability improvement (positive and significant coefficient in columns 4 and 6). Finally, coefficients for income gains (“IncGain_SCS” and “IncGain_waves”) are positive and significant in column 4.

From Table 17 we learn that income losses and income gains are important drivers of make ends meet variations. Tables 18 and 19 report summary statistics for income losses and gains. The tables differ for the sample in use. While Table 18 reports statistics over the full sample, Table 19 restricts to the subsample of households in which the same respondent provided income information both in wave 7 and in the SCS. The results in the tables show that income variations (losses and gains) are more frequent between waves than during the pandemic. Income gains are more common than income losses between waves, but the opposite holds for pandemic variations. Differently from income gains (between waves and during the pandemic) and income losses between waves, income losses during the pandemic affected households differently according to their age profile. We can see in Table 18 that 25.83% of households with at least one respondent younger than 65 reported an income loss during the first wave of the pandemic, compared to 9.82% for households with all 65+ respondents. We learn from Table 18 and Table 19 that the important role played by income losses compared to income gains, on the probability of making ends meet improvement/worsening (see Table 17), is not explained by the number of households who reported income variations. It results, instead, (at least for the variations during the pandemic) from the age characteristics of the households who experienced the variations.

**Table 18:** Summary statistics (wave 7 and SCS)—income losses/gains

Outcomes	Mean (%)	SE (%)	Obs
Income loss SCS	17.52	0.38	31,227
At least one under 65	25.83	0.58	9210
All 65+	9.82	0.42	22,017
Income loss between waves	41.10	0.41	31,227
At least one under 65	40.68	0.64	9210
All 65+	41.49	0.51	22,017
Income gain SCS	5.15	0.29	31,227
At least one under 65	4.69	0.33	9210
All 65+	5.57	0.45	22,017
Income gain between waves	42.93	0.44	31,227
At least one under 65	44.21	0.65	9210
All 65+	41.74	0.54	22,017

The table shows household descriptive statistics (mean, standard error of the mean, and number of observations), using calibrated cross-sectional household weights and multiple imputations, of income losses and gains between wave 7 and the SCS, and during the first wave of the pandemic. We consider an income loss/gain when the income variation is 5% or greater. Income loss SCS and Income gain SCS result from the difference between the lowest household income during the pandemic and the typical household income before COVID-19 broke out. Income loss waves and Income gain waves, instead, are the indicators for losses and gains resulting from the comparison of the typical household income before COVID-19 broke out and the typical household income in wave 7

*Source* Authors’ elaboration using SCS and SHARE waves 1, 2, 4, 5, 6, 7, and 8 data

**Table 19:** Summary statistics (wave 7 and SCS)—income losses/gains, same respondent

Outcomes	Mean (%)	SE (%)	Obs
Income loss SCS	17.05	0.43	25,569
At least one under 65	25.49	0.66	7175
All 65+	9.85	0.47	18,394
Income loss between waves	41.32	0.45	25,569
At least one under 65	41.03	0.68	7175
All 65+	41.57	0.52	18,394
Income gain SCS	5.54	0.35	25,569
At least one under 65	5.19	0.59	7175
All 65+	5.83	0.45	18,394
Income gain between waves	42.97	0.48	25,569
At least one under 65	44.16	0.78	7175
All 65+	41.95	0.52	18,394

The table shows household descriptive statistics (mean, standard error of the mean, and number of observations), using calibrated cross-sectional household weights and multiple imputations, of income losses and gains between wave 7 and the SCS, and during the first wave of the pandemic. The table reports summary statistics of income losses and gains, for households in which the same respondent provided income information both in wave 7 and in the SCS. We consider an income loss/gain when the income variation is 5% or greater. Income loss SCS and Income gain SCS result from the difference between the lowest household income during the pandemic and the typical household income before COVID-19 broke out. Income loss waves and Income gain waves, instead, are the indicators for losses and gains resulting from the comparison of the typical household income before COVID-19 broke out and the typical household income in wave 7

*Source* Authors’ elaboration using SCS and SHARE waves 1, 2, 4, 5, 6, 7, and 8 data

### 6.4

In this section we extend our investigation on the determinants of different measures of financial distress that we discussed in Sect. 4 and Appendix 6.3. In Tables 20, 21, and 22 we present OLS estimation results when including additional employment characteristics: last job industry (the kind of business, industry, or services of the last job) and last job type (private-sector employee, public sector employee, or self-employed).^18^ We refer the reader to footnotes 11 and 12 for more details about the controls.

**Table 20:** OLS regressions—dependent variables: difficulties in make ends meet, postpone payments, dip into savings, income loss, and financial distress indicator

	MeM_SCS_	Postpay	DipSav	IncLoss_SCS_	FDI
Female	− 0.002	− 0.005	− 0.004	− 0.010	− 0.014
	(0.006)	(0.009)	(0.012)	(0.007)	(0.010)
Household size	0.021***	0.012***	0.003	0.013***	0.042***
	(0.003)	(0.003)	(0.004)	(0.003)	(0.005)
*HH composition and age*					
single ≥ 65	− 0.081***	− 0.060***	− 0.032**	− 0.038***	− 0.154***
	(0.009)	(0.012)	(0.014)	(0.009)	(0.015)
couple < 65	0.023**	0.000	− 0.009	− 0.051***	− 0.032*
	(0.011)	(0.014)	(0.017)	(0.012)	(0.019)
couple ≥ 65	− 0.036***	− 0.052***	− 0.057***	− 0.075***	− 0.138***
	(0.010)	(0.013)	(0.016)	(0.010)	(0.017)
Years of education	− 0.004***	0.001	0.007***	0.001	− 0.003**
	(0.001)	(0.001)	(0.001)	(0.001)	(0.001)
Employed	0.006	− 0.012	0.017	0.030	0.039
	(0.016)	(0.027)	(0.034)	(0.019)	(0.028)
Private employee	− 0.006	0.048	0.077*	0.038*	0.046
	(0.018)	(0.037)	(0.043)	(0.023)	(0.034)
Public employee	− 0.017	− 0.010	0.013	0.011	− 0.007
	(0.018)	(0.038)	(0.044)	(0.024)	(0.035)
Self-employed	0.046**	0.142***	0.204***	0.119***	0.219***
	(0.019)	(0.038)	(0.046)	(0.025)	(0.037)
*Industries*					
Agriculture, etc.	− 0.035	− 0.080**	− 0.165***	− 0.006	− 0.076*
	(0.023)	(0.038)	(0.049)	(0.028)	(0.043)
Mining and quarrying	− 0.010	− 0.172***	− 0.070	0.015	− 0.045
	(0.054)	(0.037)	(0.140)	(0.072)	(0.087)
Manufacturing	− 0.022	− 0.012	− 0.017	0.013	− 0.023
	(0.018)	(0.033)	(0.042)	(0.023)	(0.034)
Electricity, gas and water	− 0.030	− 0.035	− 0.062	− 0.020	− 0.070
	(0.030)	(0.057)	(0.090)	(0.035)	(0.053)
Construction	− 0.036*	0.000	− 0.098*	− 0.010	− 0.057
	(0.021)	(0.043)	(0.052)	(0.028)	(0.041)
Wholesale and retail trade	− 0.010	− 0.042	− 0.080*	0.025	− 0.005
	(0.019)	(0.035)	(0.042)	(0.023)	(0.035)
Hotels and restaurants	0.066**	0.005	0.012	0.138***	0.217***
	(0.032)	(0.046)	(0.056)	(0.034)	(0.056)
Transport, storage, communic	0.019	− 0.047	− 0.049	0.019	0.024
	(0.021)	(0.038)	(0.047)	(0.024)	(0.038)
Financial intermediation	− 0.010	− 0.019	0.001	− 0.026	− 0.058
	(0.024)	(0.064)	(0.087)	(0.030)	(0.043)
Real estate, renting, businesses	0.038	0.088	0.076	0.011	0.052
	(0.029)	(0.070)	(0.078)	(0.039)	(0.059)
Public admin. and defence	− 0.023	0.014	− 0.040	− 0.019	− 0.048
	(0.020)	(0.042)	(0.051)	(0.025)	(0.039)
Education	− 0.022	− 0.017	− 0.078*	− 0.025	− 0.062*
	(0.019)	(0.039)	(0.047)	(0.023)	(0.036)
Health and social work	0.007	− 0.025	− 0.074*	− 0.009	− 0.021
	(0.017)	(0.033)	(0.043)	(0.022)	(0.032)
Other community	0.008	− 0.016	− 0.068	0.024	0.025
	(0.017)	(0.033)	(0.041)	(0.024)	(0.034)
Job interruption	− 0.021	0.128***	0.193***	0.196***	0.212***
	(0.022)	(0.037)	(0.046)	(0.028)	(0.044)
Home working	− 0.012	0.024	− 0.032	− 0.043***	− 0.056***
	(0.010)	(0.021)	(0.026)	(0.011)	(0.018)
Reduced working hours	0.019*	0.015	0.030	0.193***	0.212***
	(0.011)	(0.021)	(0.028)	(0.015)	(0.022)
Weeks of job interruption	0.012***	0.000	0.003	0.015***	0.030***
	(0.002)	(0.003)	(0.004)	(0.003)	(0.004)
Income from others	0.010*	0.006	0.002	0.011**	0.023***
	(0.006)	(0.007)	(0.010)	(0.005)	(0.009)
Second homes	− 0.010*	− 0.004	0.011	0.001	− 0.010
	(0.005)	(0.008)	(0.010)	(0.005)	(0.008)
Investments	− 0.032***	− 0.009	0.008	− 0.004	− 0.037***
	(0.005)	(0.012)	(0.019)	(0.006)	(0.009)
Own business	0.025***	0.020	0.045*	0.033***	0.063***
	(0.009)	(0.016)	(0.023)	(0.010)	(0.016)
Tenant	0.049***	0.050***	− 0.000	0.005	0.072***
	(0.007)	(0.010)	(0.013)	(0.006)	(0.010)
log(Income before COVID-19)	− 0.224***	− 0.056***	− 0.007	0.024***	− 0.234***
	(0.007)	(0.008)	(0.010)	(0.005)	(0.010)
Length of restrictions	0.002	− 0.006***	− 0.004	− 0.000	− 0.000
	(0.001)	(0.002)	(0.002)	(0.001)	(0.002)
Avg. restriction intensity	0.000	− 0.007	− 0.000	0.001	0.000
	(0.004)	(0.005)	(0.008)	(0.004)	(0.006)
MeM wave7	0.247***	0.029***	0.015*	0.013***	0.284***
	(0.006)	(0.006)	(0.009)	(0.005)	(0.009)
Observations	31,060	10,474–10,514	10,474–10,514	31,060	31,060
*R*^2^	0.398	0.088	0.113	0.178	0.341

Dependent variables are binary indicators for: (some or great) difficulties in making ends meet during the pandemic (column 1), postpone payment (column 2), dip into savings (column 3), income losses during the pandemic (column 4), and Financial Distress Indicator. The results obtained using multiple imputation technique for missing values. Note that since the variables postpone payment (Postpay) and dip into savings (DipSav) are collected conditional on reporting some or great difficulties in making ends meet, imputations are computed only when the condition is met, and estimation subsample vary across imputations in columns 2 and 3. Robust standard errors in parentheses. ****p * < 0.01; ***p* < 0.05; **p* < 0.1

*Source* Authors’ elaboration using SCS and SHARE waves 1, 2, 4, 5, 6, 7, and 8 data

**Table 21:** OLS regressions—Dependent variables: Income loss during the pandemic, percentage income variation during the pandemic

	Income loss (SCS)			Percentage income loss (SCS)	
	(full sample)	JI or RWH	No JI and No RWH	JI or RWH	No JI and No RWH
Female	− 0.010	− 0.040	− 0.009	− 18.409	− 0.040
	(0.007)	(0.036)	(0.007)	(18.246)	(0.584)
Household size	0.013***	0.029**	0.013***	1.131	0.215
	(0.003)	(0.012)	(0.003)	(2.382)	(0.292)
*HH composition and age:*					
single ≥ 65	− 0.038***	0.015	− 0.039***	0.124	2.106**
	(0.009)	(0.043)	(0.010)	(11.352)	(0.853)
Couple < 65	− 0.051***	− 0.107***	− 0.043***	24.853*	5.795***
	(0.012)	(0.036)	(0.013)	(12.943)	(1.817)
Couple ≥ 65	− 0.075***	− 0.128***	− 0.071***	24.180**	6.735***
	(0.010)	(0.043)	(0.010)	(10.096)	(1.844)
Years of education	0.001	0.003	0.000	1.642	0.079
	(0.001)	(0.004)	(0.001)	(1.462)	(0.087)
Employed	0.030		0.020		− 0.147
	(0.019)		(0.022)		(1.284)
Private employee	0.038*	0.100**	0.014	− 6.605	0.016
	(0.023)	(0.046)	(0.027)	(4.602)	(1.223)
Public employee	0.011	0.020	0.003	1.710	1.764
	(0.024)	(0.047)	(0.028)	(4.142)	(1.606)
Self-employed	0.119***	0.178***	0.073**	− 6.574	− 3.047**
	(0.025)	(0.046)	(0.029)	(6.574)	(1.385)
*Industries*					
	− 0.006	0.016	0.016	− 0.798	0.468
	(0.028)	(0.060)	(0.032)	(6.019)	(1.275)
Mining and quarrying	0.015	− 0.043	0.034	14.702	1.277
	(0.072)	(0.172)	(0.077)	(10.943)	(2.236)
Manufacturing	0.013	0.007	0.022	12.452*	1.499
	(0.023)	(0.041)	(0.026)	(7.465)	(1.212)
Electricity, gas and water	− 0.020	− 0.030	− 0.008	13.617	1.219
	(0.035)	(0.077)	(0.037)	(8.612)	(2.032)
Construction	− 0.010	− 0.009	0.003	15.016	3.057
	(0.028)	(0.047)	(0.032)	(10.178)	(2.835)
Wholesale and retail trade	0.025	0.031	0.015	20.038	4.444
	(0.023)	(0.041)	(0.027)	(15.717)	(3.114)
Hotels and restaurants	0.138***	0.115***	0.108*	2.498	0.671
	(0.034)	(0.044)	(0.059)	(6.825)	(5.289)
Transport, storage, communic	0.019	0.052	0.011	3.977	1.907
	(0.024)	(0.048)	(0.029)	(6.291)	(1.460)
Financial intermediation	− 0.026	− 0.039	− 0.018	12.474	2.164
	(0.030)	(0.065)	(0.031)	(8.615)	(1.731)
Real estate, renting, business act	0.011	− 0.025	0.031	10.062	1.959
	(0.039)	(0.066)	(0.047)	(7.646)	(1.469)
Public admin. and defence	− 0.019	− 0.052	− 0.004	7.689	1.483
	(0.025)	(0.054)	(0.027)	(5.405)	(1.463)
Education	− 0.025	− 0.088**	0.002	11.660*	0.698
	(0.023)	(0.043)	(0.029)	(6.074)	(1.504)
Health and social work	− 0.009	− 0.004	− 0.001	10.770	1.127
	(0.022)	(0.040)	(0.024)	(8.980)	(1.374)
Other community	0.024	0.019	0.027	3.577	− 0.048
	(0.024)	(0.040)	(0.027)	(5.014)	(1.204)
Job interruption	0.196***				
	(0.028)				
Home working	− 0.043***	− 0.068***	− 0.014	12.894	1.616**
	(0.011)	(0.025)	(0.013)	(10.237)	(0.804)
Reduced working hours	0.193***				
	(0.015)				
Weeks of job interruption	0.015***	0.014***		− 1.160***	
	(0.003)	(0.002)		(0.351)	
Income from others	0.011**	− 0.008	0.013**	− 3.076	− 0.387
	(0.005)	(0.026)	(0.005)	(3.390)	(0.610)
Second homes	0.001	0.007	0.001	− 3.002	− 0.190
	(0.005)	(0.023)	(0.005)	(2.523)	(0.507)
Investments	− 0.004	− 0.021	− 0.003	10.040	0.391
	(0.006)	(0.027)	(0.006)	(11.101)	(0.635)
Own business	0.033***	0.039	0.033***	1.848	− 1.837**
	(0.010)	(0.032)	(0.011)	(5.473)	(0.825)
Tenant	0.005	0.034	0.001	13.135	− 1.297**
	(0.006)	(0.032)	(0.006)	(15.405)	(0.548)
log(Income before COVID-19)	0.024***	0.053**	0.020***	− 59.661	− 10.622***
	(0.005)	(0.026)	(0.005)	(44.651)	(3.468)
Length of restrictions	− 0.000	0.003	0.000	0.278	0.180
	(0.001)	(0.006)	(0.001)	(0.791)	(0.199)
Avg. restriction intensity	0.001	− 0.005	0.001	− 5.818	0.832
	(0.004)	(0.022)	(0.004)	(4.729)	(0.617)
MeM wave7	0.013***	0.032	0.011***	− 13.278	− 2.449***
	(0.005)	(0.023)	(0.004)	(9.825)	(0.778)
Observations	31,060	2514–2529	28,531–28,546	2514–2529	28,531–28,546
*R*^2^	0.178	0.142	0.052	0.088	0.021

Dependent variables in columns 1–3 are binary indicators for income losses during the pandemic. Column 1 refers to the entire sample. Column 2 focuses on the subsample of households that reported either a “Job interruption” (JI) or “Reduced Working Hours” (RWH). Column 3 refers to the subsample of households that did not have “Job interruption” (JI) or “Reduced Working Hours” (RWH). Columns 4 and 5 focus on the same subsamples of columns 2 and 3 but use as dependent variables income variations, in percentage terms, during the pandemic (they span from negative to positive values). The results obtained using multiple imputation technique for missing values. Note that since the variables job interruption and reduction in working hours are collected conditional on being employed, imputations are computed only when the condition is met, and estimation subsample vary across imputations in columns 2–5. Robust standard errors in parentheses. ****p * < 0.01; ***p* < 0.05; **p* < 0.1

*Source* Authors’ elaboration using SCS and SHARE waves 1, 2, 4, 5, 6, 7, and 8 data

**Table 22:** OLS regressions—Dependent variables: worsening and improvement in making ends meet

	MeM worsening			MeM improvement		
		(Income loss)	(No income loss)		(Income gain)	(No income gain)
Female	− 0.008	− 0.014	− 0.001	− 0.017	− 0.015	− 0.017
	(0.007)	(0.012)	(0.008)	(0.013)	(0.018)	(0.018)
Household size	0.016***	0.006	0.021***	− 0.023***	− 0.033***	− 0.016***
	(0.004)	(0.006)	(0.006)	(0.005)	(0.007)	(0.006)
plusDeltaSize	− 0.002	0.016	− 0.015	− 0.006	− 0.018	0.026
	(0.024)	(0.037)	(0.030)	(0.028)	(0.038)	(0.041)
minDeltaSize	0.021**	0.015	0.013	0.011	− 0.010	0.018
	(0.009)	(0.012)	(0.013)	(0.013)	(0.023)	(0.018)
*HH composition and age*						
single ≥ 65	− 0.075***	− 0.092***	− 0.043***	0.071***	0.066***	0.082***
	(0.013)	(0.019)	(0.016)	(0.015)	(0.021)	(0.021)
couple < 65	0.000	0.005	0.007	− 0.051***	− 0.062**	− 0.033
	(0.013)	(0.020)	(0.017)	(0.017)	(0.024)	(0.025)
couple ≥ 65	− 0.039***	− 0.049**	− 0.015	0.020	0.011	0.035
	(0.013)	(0.020)	(0.017)	(0.017)	(0.024)	(0.025)
Years of education	− 0.004***	− 0.005***	− 0.002**	0.003***	0.003	0.004**
	(0.001)	(0.001)	(0.001)	(0.001)	(0.002)	(0.002)
*Employment variation*						
Always employed	− 0.005	− 0.001	0.004	− 0.010	0.003	− 0.041
	(0.018)	(0.031)	(0.021)	(0.033)	(0.044)	(0.058)
Employed-Not empl	0.013	0.011	0.005	− 0.012	− 0.020	− 0.018
	(0.010)	(0.014)	(0.014)	(0.017)	(0.029)	(0.021)
Not empl—Employed	0.026	0.043	0.014	− 0.011	− 0.009	− 0.000
	(0.020)	(0.033)	(0.023)	(0.034)	(0.043)	(0.063)
Private employee	− 0.018	− 0.009	− 0.025	− 0.027	− 0.025	− 0.042
	(0.020)	(0.032)	(0.021)	(0.038)	(0.049)	(0.059)
Public employee	− 0.035*	− 0.034	− 0.034*	− 0.033	− 0.028	− 0.052
	(0.019)	(0.032)	(0.020)	(0.039)	(0.051)	(0.064)
Self-employed	0.021	0.036	0.001	− 0.068*	− 0.061	− 0.099
	(0.021)	(0.033)	(0.022)	(0.041)	(0.056)	(0.061)
*Industries*						
Agriculture, etc.	− 0.010	− 0.063	0.037	0.076*	0.062	0.120
	(0.026)	(0.042)	(0.032)	(0.043)	(0.054)	(0.075)
Mining and quarrying	− 0.038	− 0.154***	0.135	− 0.039	− 0.055	0.071
	(0.064)	(0.056)	(0.133)	(0.098)	(0.114)	(0.198)
Manufacturing	0.004	− 0.019	0.022	0.085**	0.067	0.122*
	(0.020)	(0.033)	(0.021)	(0.038)	(0.048)	(0.066)
Electricity, gas and water	0.008	0.004	− 0.001	0.141**	0.074	0.257**
	(0.030)	(0.050)	(0.032)	(0.069)	(0.093)	(0.110)
Construction	0.008	− 0.016	0.029	0.128***	0.137**	0.114
	(0.022)	(0.037)	(0.024)	(0.044)	(0.056)	(0.072)
Wholesale and retail trade	− 0.011	− 0.048	0.029	0.018	0.023	0.022
	(0.021)	(0.034)	(0.027)	(0.039)	(0.052)	(0.058)
Hotels and restaurants	0.071*	0.054	0.051	− 0.020	− 0.102	0.114
	(0.041)	(0.052)	(0.070)	(0.055)	(0.071)	(0.089)
Transport, storage, communic	0.053**	0.070*	0.025	0.067	0.036	0.122*
	(0.023)	(0.038)	(0.027)	(0.043)	(0.055)	(0.073)
Financial intermediation	0.019	− 0.004	0.029	0.110	0.106	0.086
	(0.023)	(0.041)	(0.024)	(0.069)	(0.087)	(0.111)
Real estate, renting, business act	0.046	0.025	0.056*	0.002	− 0.016	0.045
	(0.030)	(0.050)	(0.033)	(0.081)	(0.102)	(0.141)
Public admin. and defence	0.017	− 0.004	0.030	0.113**	0.080	0.184**
	(0.020)	(0.035)	(0.022)	(0.044)	(0.059)	(0.076)
Education	0.011	− 0.005	0.013	0.098**	0.139**	0.040
	(0.020)	(0.034)	(0.021)	(0.043)	(0.056)	(0.066)
Health and social work	0.022	0.002	0.036*	0.030	0.024	0.053
	(0.018)	(0.031)	(0.019)	(0.039)	(0.050)	(0.067)
Other community	0.025	0.025	0.014	0.035	0.029	0.060
	(0.019)	(0.033)	(0.021)	(0.036)	(0.046)	(0.058)
Job interruption		− 0.032	− 0.082**	− 0.027	− 0.028	− 0.002
		(0.035)	(0.034)	(0.041)	(0.057)	(0.059)
Home working	− 0.013	− 0.012	− 0.013	0.049**	0.021	0.096**
	(0.010)	(0.016)	(0.011)	(0.025)	(0.031)	(0.041)
Reduced working hours	− 0.004	− 0.007	0.024	− 0.027	− 0.014	− 0.046
	(0.012)	(0.018)	(0.015)	(0.024)	(0.032)	(0.039)
Weeks of job interruption	0.006***	0.010***	0.010**	− 0.011***	− 0.013**	− 0.011*
	(0.002)	(0.003)	(0.005)	(0.004)	(0.005)	(0.006)
IncLoss_SCS	0.092***	0.073***				− 0.001
	(0.011)	(0.019)				(0.018)
IncLoss_waves	0.019***	− 0.018				0.031**
	(0.006)	(0.021)				(0.013)
IncGain_SCS			0.030	0.049**	0.051*	
			(0.025)	(0.019)	(0.030)	
IncGain_waves			− 0.002	0.021**	− 0.005	
			(0.007)	(0.009)	(0.029)	
log(Income before COVID-19)	− 0.170***	− 0.194***	− 0.146***	0.272***	0.301***	0.250***
	(0.008)	(0.012)	(0.010)	(0.011)	(0.018)	(0.015)
Income from others	0.009	0.011	0.006	− 0.011	− 0.012	− 0.014
	(0.006)	(0.010)	(0.008)	(0.010)	(0.014)	(0.016)
Second homes	− 0.012**	− 0.009	− 0.012*	0.011	0.006	0.017
	(0.006)	(0.009)	(0.007)	(0.010)	(0.015)	(0.015)
Investments	− 0.023***	− 0.035***	− 0.012**	0.089***	0.085***	0.096***
	(0.005)	(0.008)	(0.006)	(0.017)	(0.024)	(0.027)
Own business	0.026***	0.040***	0.010	− 0.037*	− 0.036	− 0.045
	(0.010)	(0.014)	(0.012)	(0.021)	(0.030)	(0.031)
Tenant	0.032***	0.045***	0.019**	− 0.080***	− 0.100***	− 0.048**
	(0.008)	(0.013)	(0.009)	(0.012)	(0.017)	(0.020)
Length of restrictions	0.003*	0.003	0.002	− 0.001	0.001	− 0.005
	(0.002)	(0.002)	(0.002)	(0.003)	(0.004)	(0.004)
Avg. restriction intensity	0.001	0.005	− 0.005	0.003	0.005	− 0.001
	(0.004)	(0.007)	(0.006)	(0.008)	(0.012)	(0.013)
Observations	18,264	8771–8815	9449–9493	12,796	7252–7306	5490–5544
*R*^2^	0.200	0.219	0.146	0.238	0.222	0.235

Dependent variables in columns 1, 2 and 3 are binary indicators for worsening in making ends meet from wave 7 to SCS, for the subsample of households that did not report some or great difficulties in making ends meet in wave7. Dependent variables in Columns 4, 5 and 6, instead, are binary indicators for improvement in making ends meet from wave 7 to SCS, for the subsample of households who had some or great difficulties in wave7. Columns 1 and 4 refer to the full (respective) subsamples. Columns 2 and 5 focus, respectively, on the subsample of households with an income loss or an income gain (either between waves or during the pandemic), while columns 3 and 6 focuses, respectively, on the subsample of households without any income loss or income gain. The results obtained using multiple imputation technique for missing values. Note that since the variables income loss and income gains depend on income values either reported by the respondents or imputed, estimation samples vary across imputations in columns 2–3 and 5–6. Robust standard errors in parentheses. ****p * < 0.01; ***p* < 0.05; **p* < 0.1

*Source* Authors’ elaboration using SCS and SHARE waves 1, 2, 4, 5, 6, 7, and 8 data

The results in all three tables show that our previous findings are robust to the inclusion of information on last job industry and type. Focusing on the last job type in Table 20 (correspondent regressions in Tables 7 and 15), we can see that being “Self-employed” significantly increases financial distress, the probability of having some or great difficulties in making ends meet, the probability to suffer from income losses during the pandemic, and the probabilities to dip into savings and postpone payments.

From Table 22, instead, we learn that if at least one respondent worked in “Transport, storage, communic.” this increases the probability of a make ends meet ability deterioration. Working in “Manufacturing”, “Electricity, gas and water”, “Construction”, “Public admi. and defence”, and “Education”, instead, has a positive effect on the probability of a make ends meet improvement.

#### Country-specific policy reactions to the pandemic and economic outcomes

To contain the spread of COVID-19, governments adopted a series of measures ranging from mild mobility restrictions to strict lockdown of economic activities, following the evolution of the pandemic. Such restrictions in many cases exacerbated (sometimes pre-existing) household economic difficulties.

Figure 3 shows, for each country, the percentage of households reporting a worsening of their ability to make ends meet (from no difficulties to some or great difficulties) against the OxCGRT “stringency index”.

Restriction severity ranges from a minimum of 40.75% for Estonia to a maximum of 63.89% for Italy, with an average value of 54.85%.^19^ The intensity of government restrictive measures is especially high (above the average) in most of the Mediterranean countries (Spain, Italy, France, Portugal, and Cyprus) together with Romania and Israel.

Across countries, the average percentage of households that are worse off (in terms of making ends meet) is about 8.50%. Countries that report great worsening are in South and East Europe (Spain, Italy, Cyprus, Poland, Hungary, Slovenia, Estonia, Croatia, Lithuania, Bulgaria, Latvia, Malta, Romania, and Slovakia plus Israel).

Figure 3 shows a positive, although weak, correlation between household economic distress and severity of restrictions. We can see that eleven countries lie above the regression line (Estonia, Latvia, Bulgaria, Slovenia, Slovakia, Hungary, Malta, Poland, Romania, Spain, and Israel), indicating a marked worsening in making ends meet with respect to what we can predict from the stringency index, while the remaining sixteen countries are on or below the line.

## Footnotes

1 For both the lowest household income during the pandemic and the household typical income before the pandemic, respondents were asked to provide the amount of the overall monthly income, after taxes and contributions, for the entire household. For the former income, the lowest during the pandemic, respondents were requested to include any financial support they may have received since the outbreak of the pandemic.

2 The questions on the employment situation before the pandemic ask the respondent his situation at the time of the interview (wave 7) or at the time when COVID-19 broke out (SCS). Employed respondents include employees or self-employed, and those working in a family business.

3 The economic section of the questionnaire was asked only to the first respondent in the household. The entire section is controlled by the variable CAE001. However, the system did not automatically assign the filter variable, which instead was coded by the interviewer/respondent. This led to routing errors: some households had two first respondents (therefore two valid answers for all the variables in the economic section), while some of them have none (therefore they skipped the economic section). We followed the strategy adopted by SHARE and described in the SHARE Corona Survey Release 8.0.0 and recoded the variable CAE001. We started with 39,291 first respondents and ended up with 39,104 first respondents. See the SHARE Corona Survey Release 8.0.0 for more details on the adopted strategy. http://www.share-project.org/fileadmin/pdf_documentation/SHARE_Corona_Survey_Release_Guide.pdf.

4 We drop 7877 households. For 5127 of them (65.09%) we miss information about the employment situation of the household members in wave 7. For other 2098 households (26.63%) we miss data on ownership of the main residence (owner or tenant of the dwelling the household lives in). For the remaining households we miss (in descending order) information about financial investments, income from others, ability to make ends meet in wave 7, typical household income in wave 7, SCS calibrated cross-sectional household weights, and owning a business.

5 Estimates from the imputed datasets were pooled using the functions misum, mim, mi estimate and mibeta in Stata (Klein 2011; Galati et al. 2013; Harel 2009; Marchenko 2010; StataCorp 2021).

6 The stringency index measures, using a score between 0 and 100, the strictness of ‘lockdown style’ closures and containment policies adopted by governments.

7 We label as ‘employed’ those households with at least one employed household respondent at the time COVID-19 broke out.

8 We refer the reader to Appendix 6.2 for more details on employment and income losses.

9 See Sect. 3.2 for more details on the Financial Distress Indicator.

10 Different model specifications (Probit for MeM_SCS_, MeM_worsening, and MeM_improvement and Ordered Probit for FDI) produce similar results.

11 Reference country for country dummies: Germany. “Female” is a dummy variable that equals one if there is at least one female respondent. “Household size” takes values from 1 to 11, while for “HH composition and age” the reference category is single under 65. “Years of education” equals 5, 8, 13, 15, and 17 for, respectively, primary, lower secondary, upper secondary, post-secondary, and tertiary maximum level of education among household respondents. “Employed”, “Job interruption”, “Home working”, and “Reduced working hours” are dummy variables that take value 1 if at least one household respondent, respectively, was employed at the time COVID-19 broke out, experienced at least one job interruption, worked (at least partly) from home, and reduced working hours (but did not have any job interruption) during the pandemic. “Weeks of job interruption” is a continuous variable reflecting the number of weeks of job interruption. “Income from others”, “Second homes”, “Investments”, “Own business”, and “Tenant” are dummy variables that equal one if the household receives income from other household members (different from the respondents), owns housing stock that is not used as main residence, owns financial investments, owns businesses, and occupies the dwelling they live in as tenant or subtenant. “log(Income before COVID-19)” is the inverse hyperbolic sine of the income. “Length of restrictions” and “Avg. restriction intensity” are the length and average intensity of restrictions derived from the OxCGRT “stringency index”. To measure restriction severity, we compute, for each household, the average country-level stringency index for the time window starting from the first week in which restrictions were introduced to the week of the interview. Similarly, we measure the length of the restrictions as the number of weeks from the first week in which restrictions were introduced in the country to the week of the interview. “Make-ends-meet wave 7” is a dummy variable that equals 1 if the household reported some or great difficulties in wave 7, and 0 otherwise.

12 For details on country dummies, “Female”, “Household size”, “HH composition and age”, “Years of education”, “Job interruption”, “Home working”, “Weeks of job interruption”, “log(Income before COVID-19)”, “Income from others”, “Second homes”, “Investments”, “Own business”, “Tenant”, “Length of restrictions”, and “Avg. restriction intensity” see footnote 11 (parameter values at the time of the SCS). “plusDeltaSize” and “minDeltaSize” are dummy indicators for positive and negative variation in household size between waves. For “Employment variation” the reference category is always not employed. “IncLoss_SCS” and “IncLoss_waves” are dummy variables that take a value equal to one if the household experienced an income loss of at least 5%, respectively, during the pandemic, or between wave 7 and the SCS. “InGain_SCS” and “InGain_waves” are the correspondent dummy indicators for income gains. We remind the reader that we record as income losses/gains only income variations of at least 5%.
